# Biomimetic nanoparticles to enhance the reverse cholesterol transport for selectively inhibiting development into foam cell in atherosclerosis

**DOI:** 10.1186/s12951-023-02040-9

**Published:** 2023-08-29

**Authors:** Li Zhu, Hongjiao Li, Jiyu Li, Yuan Zhong, Shuai Wu, Meng Yan, Sheng Ni, Kun Zhang, Guixue Wang, Kai Qu, Deqin Yang, Xian Qin, Wei Wu

**Affiliations:** 1 Key Laboratory for Biorheological Science and Technology of Ministry of Education, State and Local Joint Engineering Laboratory for Vascular Implants, Bioengineering College of Chongqing University, Chongqing, 400044 China; 2 School and Hospital of Stomatology, Chongqing Medical University, Chongqing, 404100 China; 3 Chongqing University, Three Gorges Hospital, Chongqing, 404000 China; 4 Jin Feng Laboratory, Chongqing, 401329 China

**Keywords:** Biomimetic nanoparticles, Atherosclerosis, Macrophage membrane, Methotrexate, Cholesterol efflux, Target delivery

## Abstract

A disorder of cholesterol homeostasis is one of the main initiating factors in the progression of atherosclerosis (AS). Metabolism and removal of excess cholesterol facilitates the prevention of foam cell formation. However, the failure of treatment with drugs (e.g. methotrexate, MTX) to effectively regulate progression of disease may be related to the limited drug bioavailability and rapid clearance by immune system. Thus, based on the inflammatory lesion “recruitment” properties of macrophages, MTX nanoparticles (MTX NPs) camouflaged with macrophage membranes (MM@MTX NPs) were constructed for the target to AS plaques. MM@MTX NPs exhibited a uniform hydrodynamic size around ~ 360 nm and controlled drug release properties (~ 72% at 12 h). After the macrophage membranes (MM) functionalized “homing” target delivery to AS plaques, MM@MTX NPs improved the solubility of cholesterol by the functionalized β-cyclodextrin (β-CD) component and significantly elevate cholesterol efflux by the loaded MTX mediated the increased expression levels of ABCA1, SR-B1, CYP27A1, resulting in efficiently inhibiting the formation of foam cells. Furthermore, MM@MTX NPs could significantly reduce the area of plaque, aortic plaque and cholesterol crystals deposition in ApoE^−/−^ mice and exhibited biocompatibility. It is suggested that MM@MTX NPs were a safe and efficient therapeutic platform for AS.

**Graphical Abstract:**

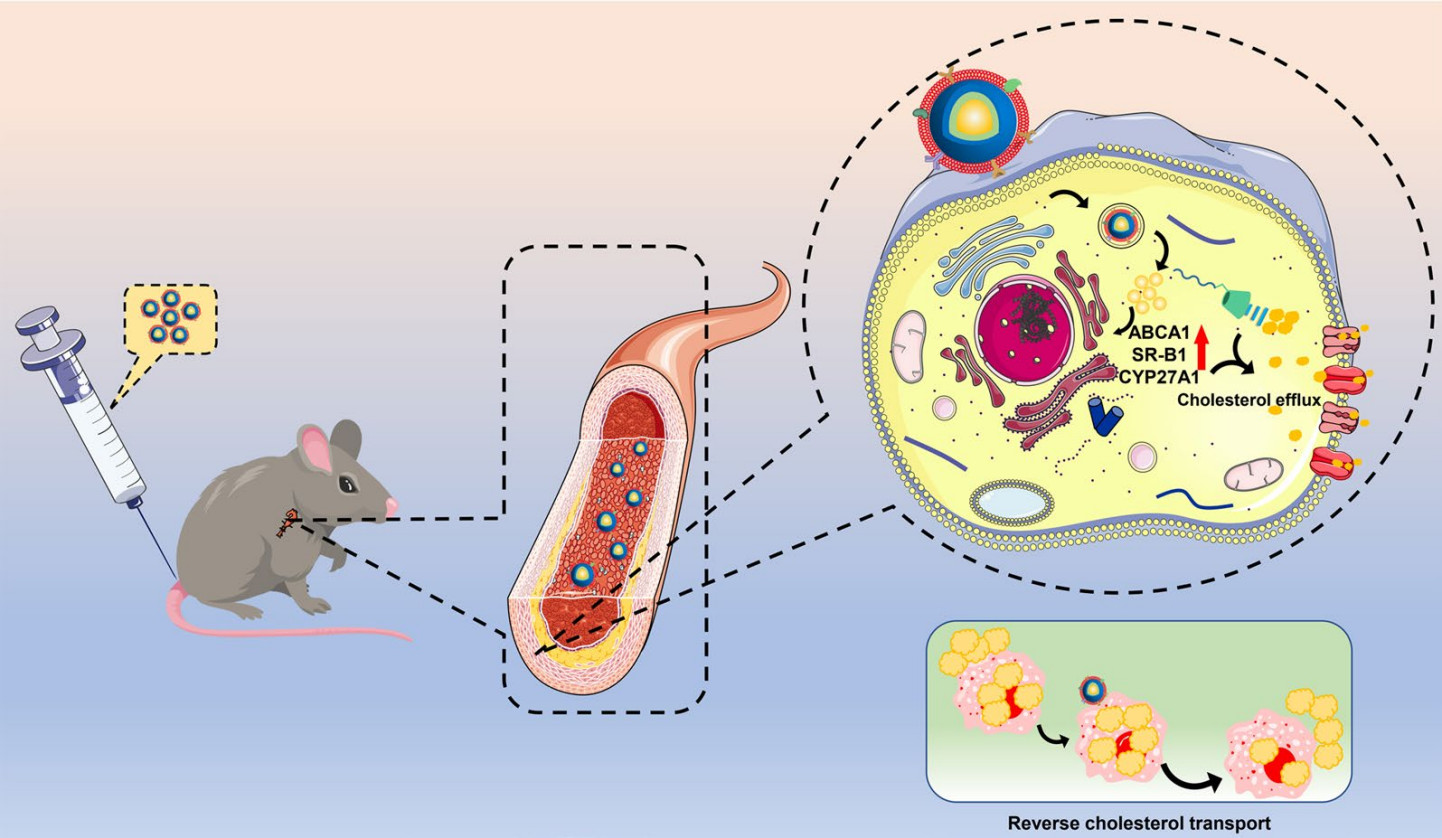

**Supplementary Information:**

The online version contains supplementary material available at 10.1186/s12951-023-02040-9.

## Introduction

Atherosclerosis (AS) as a potential risk of myocardial infarction and stroke is mainly characterized by thickening of the arterial wall due to fibrous plaque. The progression of AS is a complex process that includes factors such as inflammation and lipid accumulation. When the lipid load in the monocyte-infiltrated subendothelial muscle layer is inadequately processed under the atherosclerotic lesion, overwhelming cholesterol deposition induces macrophages and smooth muscle cells (SMCs) to become pathological foam cells while forming fatty streaks [1, 2]. Subsequently, fibrous plaques are gradually formed, reducing the arterial lumen area. Cholesterol crystal (CCs) deposition played an important role during the course of atherosclerosis [3]. Some studies have shown that CCs initially arise and accumulate in the chocolate-cell lysosomes and then are gradually deposited in atherosclerotic lesions with apoptosis of the chocolate-cells [4, 5]. CCs induces the secretion of inflammatory cytokines, such as interleukin-1β (IL-1β) and tumor necrosis factor (TNF-α), which in turn enhances monocyte infiltration and induces apoptosis in foam cells, leading to chronic inflammation [6–8].

It is well-known that only the free cholesterol can be further metabolized and intracellularly efflux by cholesterol transport proteins in lysosome, such as Niemann-Pick C-type associated protein (NPC) 1 or 2. As soon as free cholesterol has been converted to CCs, the CCs cannot be trafficked via NPC-1 or NPC-2, which results in over-loading of CCs in lysosome and consequent apoptosis [9]. Thus, preventing CCs formation or cleavage CCs from lysosomes is a widely used strategy in the clinical treatment of AS. Because of the unique molecular structure of β-Cyclodextrin (β-CD) can efficient loading or enhance cholesterol solubility through “host–guest” interactions, i.e., the β-CD lumen closely matches the cholesterol molecule [10]. Therefore, β-CD is considered the potentially effective therapeutic agent to enhance cholesterol efflux for the promising AS treatment.

Methotrexate (MTX) is an anti-inflammatory drug for the treatment of diseases such as rheumatoid arthritis and psoriatic arthritis. Recently, researchers have suggested that MTX could be effective in treating the chronic inflammatory disease AS [11, 12]. MTX has been proven to inhibit foam cell formation by MTX-induced upregulation of scavenger receptor B1 (SR-B1), ATP binding cassette transporter A1 (ABCA1) and cholesterol 27-hydroxylase (CYP27A1), which efficiently induces the cholesterol efflux [11, 13]. However, further usage of the hydrophobic MTX is greatly hindered because of the low solubility induced by the low bioavailability and the serious adverse effects in the blood [12]. Nanotechnology offers a pathway for the systematic delivery of multiple drugs. Compared to free drugs, nanomedicines can significantly increase their efficacy and reduce side effects [14–17]. Numerous studies confirm the great potential of cardiovascular disease treatment strategies based on nanomedicine [18–21]. In addition, the traditional nanoparticles (NPs) were far for the targeted drug delivery because their exogenous property will cause undesirable clearance via the immune system, which will hinder the target delivery to the pathological site [22].

Recently, cell membrane camouflage technology has provided an effective avenue for precision therapy. Through fusion between cell membranes and NPs, biomimetic NPs have the biological functions of the source cells, e.g. immune escape and self-recruitment [23]. Pei et al. [24] reported that red blood cell membrane (RBCM)-coated photosensitizer (TPC)-paclitaxel (PTX) NPs (RBCM@TPC-PTX) to synergistic chemotherapy and photodynamic therapy (PDT). The core is a PTX dimer (PTX_2_-TK) that has reactive oxygen species (ROS) responsiveness and TPC. Studies have also confirmed in vivo that RBCM@TPC-PTX can prolong circulation time, promote the accumulation of drugs and improve synergistic effectiveness of therapy at the lesion site with safe and efficient treatment. Zhao et al. [25] designed a macrophage membranes (MM)-coated Bi_2_Se_3_ NPs loaded with quercetin (M@BS-QE NPs). Based on the immune escape of MM and CC chemokine ligand 2 (CCL2)-mediated recruitment homing phenomenon, M@BS-QE NPs exhibited long-time circulation, facilitating CT and NIR-FL imaging and enhancing local accumulation of tumors.

Macrophages as a multifunctional white blood cells that are the main cells involved in inflammation response and tissue repair [26]. Previous studies suggested that MM-coating NPs have been used to treat various diseases, e.g. cancer and rheumatoid arthritis, due to their well-targeted delivery [25, 27–29]. In addition, our work suggests that the macrophage has an important effect during AS pathogenesis [21, 22, 30]. In early AS, monocytes differentiate into macrophages driven by stimulating factors and other factors. Among them, CCL2/monocyte chemoattractant protein-1 (CCL2/MCP-1) secreted from monocytes/macrophages is an important inflammatory chemokine that regulates monocyte trafficking and has been an important target for AS. CCL2/MCP-1 is capable of coordinating the inflammatory cellular transport between AS plaques by binding to the chemokine receptor protein-2 (CCR2) of macrophages [31, 32], which suggests that MM-encapsulated NPs are efficiently platform for AS target treatment.

**Fig. 1:**
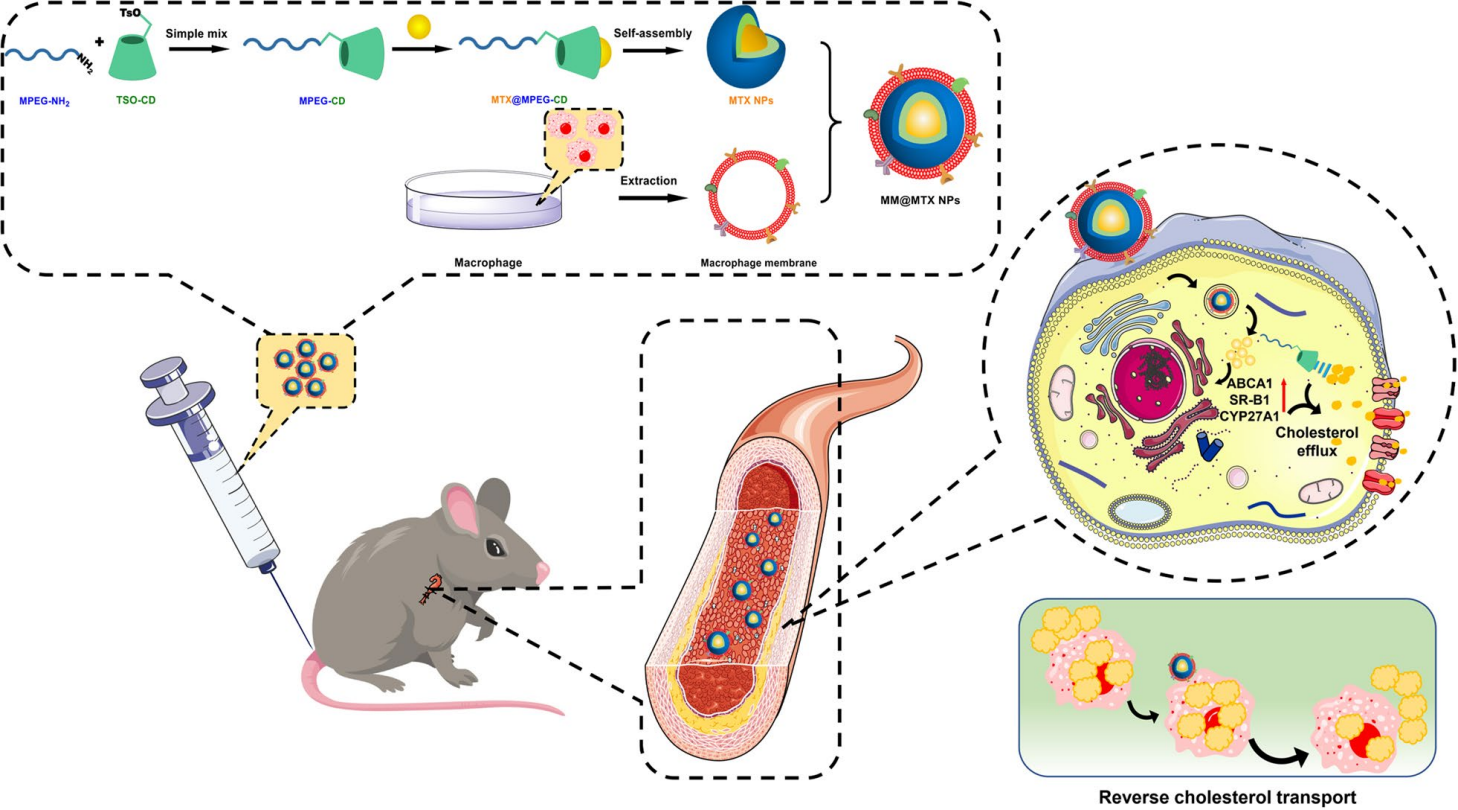
Illustrations of MM@MTX NPs enhance the reverse cholesterol transport in foam cell for the treatment of atherosclerosis

In this study, we constructed MM camouflaged NPs for the targeted treatment of AS (Fig. 1). β-CD with the powerful interaction between hydrophobic cavities and CCs has been introduced to improve the model drug MTX loading and cholesterol efflux in AS treatment. The MTX-loaded MPEGylated β-CD could be self-assembled into NPs (MTX NPs) and subsequently camouflaged with MM for targeted and effective AS administration. We hypothesized that the biomimetic MM-encapsulated MTX NPs (MM@MTX NPs) could selectively accumulate within AS plaques to enhance the local cargo release and accelerate cholesterol efflux to inhibit the progression of AS.

## Results and discussion

### Preparation and characteristics of MM@MTX NPs

**Fig. 2:**
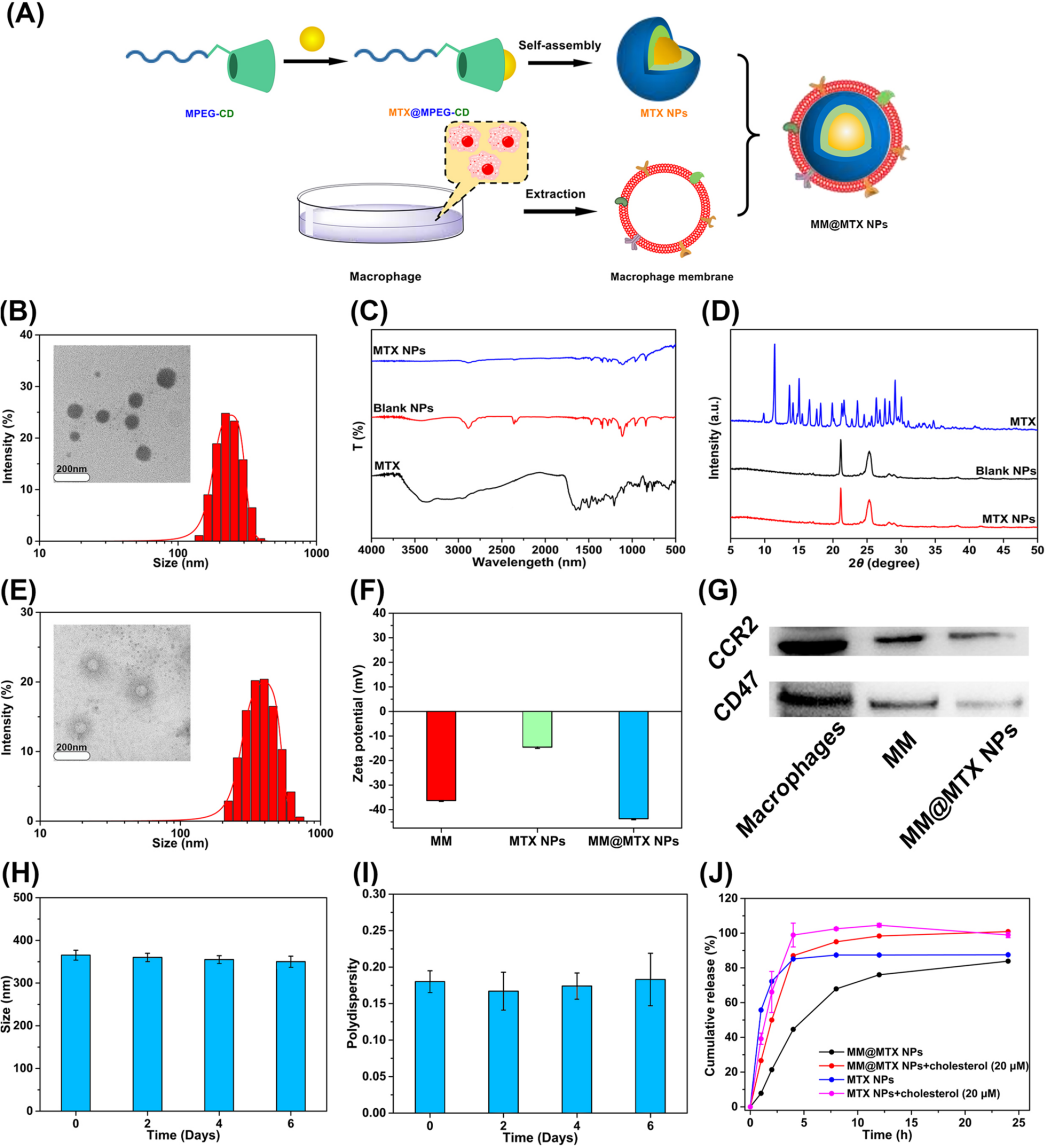
**A** The preparation diagram of MM@MTX NPs. **B** TEM and the DLS measured the size of MTX NPs (Bar = 200 nm). **C** FT-IR and **D** XRD spectrum of free MTX, blank NPs and MTX NPs. **E** TEM and the DLS measured the size of MM@MTX NPs (Bar = 200 nm). **F** Zeta potential of MM, MTX NPs and MM@MTX NPs. **G** Western blot results of CCR2 and CD47 in macrophages, MM and MM@MTX NPs. **H** Size and **I** PDI of MM@MTX NPs for 6 d in PBS (pH 7.4) (*n* = 3). **J** In vitro release of MTX from MTX NPs and MM@MTX NPs at PBS pH 7.4 with cholesterol or not, free MTX as control (*n* = 3)

According to the previous report [33], MPEG conjugated β-CD (MPEG-CD) was prepared by MPEG-NH_2_ nucleophilic substitution CD-OTs (Additional file 1: Fig. S1). ^1^H-NMR and FT-IR spectroscopy results confirmed the successful synthesis of MPEG-CD (Additional file 1: Figs. S2 and S3). Owing to its amphipathic properties, i.e., the hydrophilic PEG and the hydrophobic MTX loaded β-CD, MTX NPs could spontaneously self-assemble into NPs in aqueous solution (Fig. 2A), as shown in the result of “Tyndall effect” under the laser (Additional file 1: Fig. S4). Because of the “host–guest” inclusion of β-CD and MTX [34], DL% and EE% of MTX NPs were 6.49 ± 0.44 and 68.61 ± 5.61%, respectively. Transmission electronic microscopy (TEM) and Dynamic light scattering (DLS) analysis demonstrated that the MTX NPs particles were spherical with an average particle size of 232 ± 7.43 nm (Fig. 2B). These results confirmed that MTX-loading NPs were successfully prepared.

The binding interaction between the components was investigated by FT-IR (Fig. 2C). In spectra of free MTX, C=O peak at 1711 cm^−1^ and the primary amine -NH_2_ stretches at 3550 cm^−1^. The characteristic peak of −COOH, −CONH− and aromatic stretch for MTX were located at 1648, 1653 and 853.88 cm^−1^, respectively [35]. For blank NPs, the bands of intramolecular or intermolecularly bound primary and secondary −OH of β-CD were around 3419 cm^−1^. The C–O–C, C–H stretch could be detected at 1060 and 2887 cm^−1^, respectively. The characteristic peak of stretch appeared at the range of 1149–1114 cm^−1^ [34]. When MTX was wrapped into the NPs, the characteristic peak of –OH and C–O for β-CD were weakened and the peak of C–O–C was blue shifted to 1061 cm^−1^. The physical state of MTX in the NPs was demonstrated by XRD (Fig. 2D). The spectra of MTX show multiple crystal diffraction peaks. When MTX was encapsulated into blank NPs, the crystal diffraction peaks of MTX disappeared, indicating that MTX could include in the cavity of β-CD and showed a non-crystalline state in the NPs.

Because of the interaction between the guest and host molecules, the protons in the chemical and electronic environment change, thus the NMR signal and the chemical shift value (Δδ) can demonstrate this phenomenon [36]. ^1^H-NMR chemical shifts changes (Δδ ppm = δ complex-δ free), as shown in Additional file 1: Fig. S5 and Table S1. In Additional file 1: Table S1, H-4 (3.60–3.63 ppm, Δδ = 0.03 ppm, lower field) and H-5 (3.35–3.42 ppm, Δδ = 0.07 ppm, lower field) of MPEG-CD participated in the complexation. When the guest molecule inserted into the cavity, the protons (H-4, -5) of CD were changed and exhibited shielding/weakening impacts for NPs, resulting in a noteworthy shift from the surface protons (H-6, -7, -8, and -9) [36, 37]. The dense electron cloud at H-5 of MPEG-CD causes it to be upfield and shielded. H-4 protons show a downfield, which result from the effect of van der Waals interactions or local polarity change [36]. The ^1^H-NMR of MTX NPs was also examined. From Additional file 1: Table S1, we can observe that the chemical shifts of MTX after being loaded by MPEG-CD are mainly present in H-1 (Δ = − 0.05 ppm), H-2 (Δ = − 0.04 ppm), H-8 (Δ = − 0.15 ppm). These findings suggested that -NH_2_, -NH- is likeliest to be in the CD lumen, forming MPEG-CD@MTX complexes through intermolecular hydrogen bonding. This is also consistent with the FTIR results. To further understand the intercalation pattern in the MTX NPs. 2D NMR spectrum NOESY was performed. From Additional file 1: Fig. S6, a clear NOESY correlation can be found between MTX and the MPE-CD (H-4 and H-5), which suggested that MTX was in the CD through van der Waals or polar hydrogen bonding.

In addition, to harvest biomimetic MM@MTX NPs, native MMs were applied to the surface of MTX NPs by the extrusion method [19]. Compared with uncoated MTX NPs, the diameter of MM@MTX NPs increased to 363.1 ± 9.97 nm, which should be attributed to the additional MM coated (Fig. 2E). In addition, there is a zeta potential for the MM@MTX NPs (− 36.3 mV) corresponding to the MM (− 43.7 mV), but well above non-coated MTX NPs (− 14.5 mV) (Fig. 2F). Visual TEM images showed MM@MTX NPs showed a uniform spherical shape with a corona layer on the surface (Fig. 2E). As previously reported [38], the protein CD47 on the surface of macrophages would prevent phagocytosis by the mononuclear phagocyte system (MPS) for immune escape. Additionally, chemokine receptor CCR2 is an important factor in macrophage recruitment that could specifically recognize CCL2 and enhance targeting delivery to the inflammation lesion [39, 40]. Specific protein signaling of CCR2 was observed in macrophages cell, MM and MM@MTX NPs with Western blot measurements (Fig. 2G, Additional file 1: Fig. S7). In addition, CD47 protein was also detected. This protein is linked to SIRP-α receptor binding, which is important in regulating Macrophage phagocytosis [22]. These results indicated that the target functional proteins, CD47 and CCR2, were well retained on MM@MTX NPs (Fig. 2G). Moreover, the size and PDI of MM@MTX NPs were rarely changed in PBS (pH 7.4, 37 ℃) during 6 days investigation (Fig. 2H, I), indicating the sufficient stability for further store and applications.

The release of NPs and biomimetic NPs were investigated. At 24 h, the cumulative release ratios of MTX NPs or MM@MTX NPs were 87.54 ± 0.02 and 83.88 ± 0.05%, respectively. MM@MTX NPs showed a slightly slower MTX release rate compared to MTX NPs (Fig. 2J). The stable and long-term MTX release profile of MM@MTX NPs suggested potential applications for reducing premature cargo release during blood circulation. The binding constant between CCs and β-CD was higher than that of MTX [34, 41], which suggested CCs could enhance the release rate. When the medium has added CCs, the release rate of MTX NPs and MM@MTX NPs was significantly improved. At 8 h, the cumulative release ratios of MTX NPs and MM@MTX NPs were 102.52 ± 0.83 and 95.03 ± 0.03%, respectively. The result suggested that MM@MTX NPs could release the MTX and capture CCs at the site with high CCs levels, which would rapid the regression of AS.

### Characterization of the targeted delivery in vitro

**Fig. 3:**
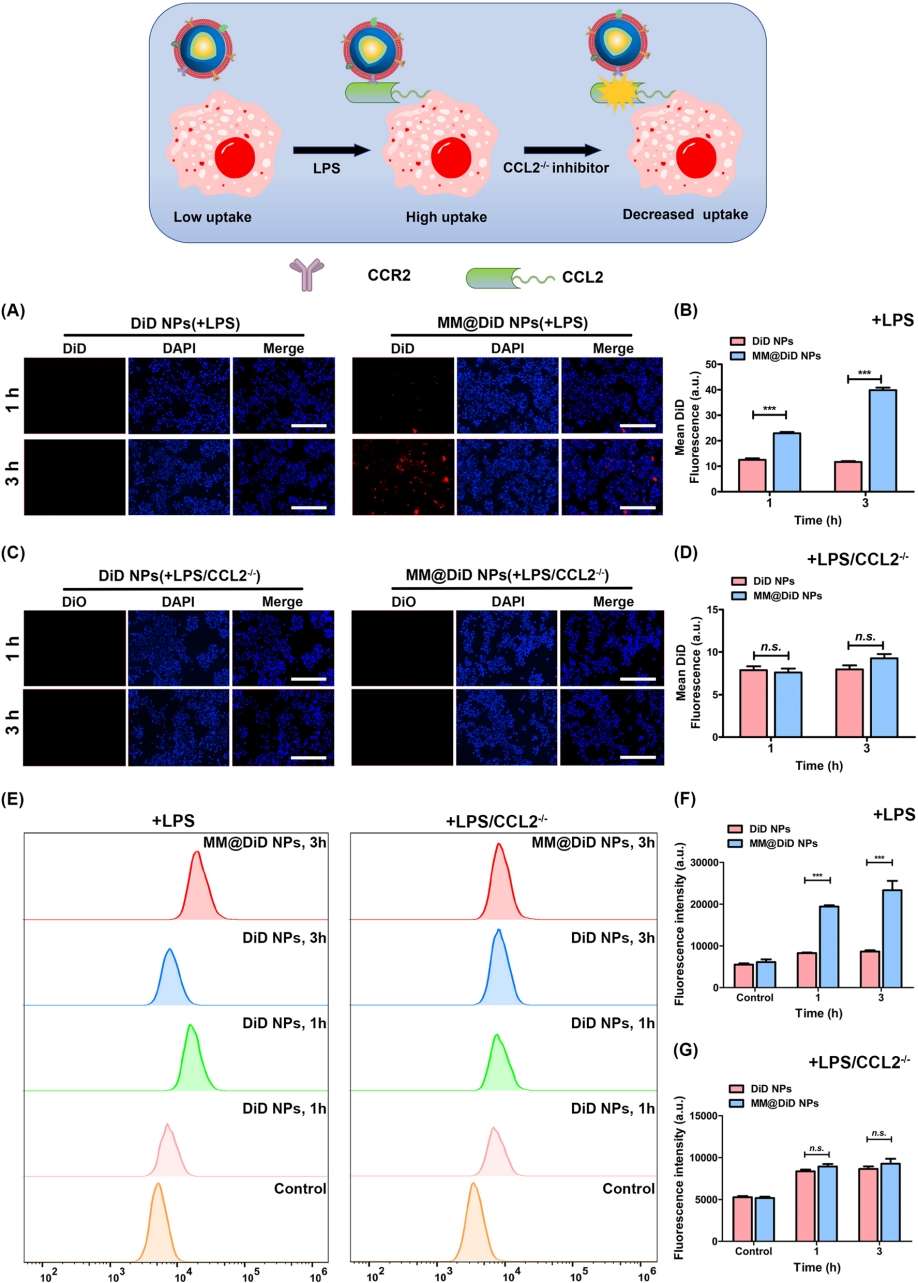
CCL2-CCR2-mediated uptake enhancement in vitro**.** Fluorescent microscopy images of **A** inflammatory macrophages and **C** CCL2-inhibited inflammatory macrophages co-cultured with DiD NPs or MM@DiD NPs (Bar = 200 μm). Quantitative uptake of DiD NPs and MM@DiD NPs by **B** inflammatory macrophages and **D** CCL2-inhibited inflammatory macrophages at 1 and 3 h (*n* = 5). **E** FCM and **D** Quantitative uptake of DiD NPs and MM@DiD NPs by **F** inflammatory macrophages and **G** CCL2-inhibited inflammatory macrophages for 1 and 3 h (*n* = 3). (***p < 0.001; *n.s.,* no significance.)

Foam cell formation plays an important role in AS [42]. Therefore, LPS-induced macrophage inflammation was chosen as the model to assess the uptake performance of MM-camouflaged biomimetic NPs. Fluorescent dye (DiD) was encapsulated into NPs (DiD NPs) and biomimetic NPs (MM@DiD NPs) to evaluate the cellular uptake behavior. From the result (Fig. 3A), no significant fluorescence in inflammatory macrophages co-cultured with DiD NPs for 1 h. In contrast, some fluorescence was detected in macrophages co-cultured with MM@DiD NPs. It was shown that MM@DiD NPs could effectively be endocytosed by the inflammatory macrophages. At 3 h, the red fluorescence signal of DiD NPs had no significant difference, whereas that of MM@DiD NPs was much stronger, as shown in the quantitative analysis of statistics (Fig. 3B). To clarify that the increased cellular uptake of MM@DiD NPs in target delivery in vitro was specificity attributed to the CCL2-CCR2 resulted from MM coating, CCL2 inhibition was selected to further investigate the cellular uptake study. As shown in Fig. 3C, the fluorescence signal of MM@DiD NPs was significantly reduced both at 1 and 3 h, and was not significantly different from that of DiD NPs, also as the quantitative analysis of the images shown (Fig. 3D). To further confirm the uptake of inflammatory macrophages, flow cytometry (FCM) was used in the study. The FCM results showed that MM@DiD NPs could enhance the uptake of inflammatory macrophages, and the fluorescence intensity of the MM@DiD NPs treated group was stronger than that of DiD NPs treated group. Whereas, after treatment by CCL2 inhibitor, the fluorescence intensity of the MM@DiD NPs treated group was significantly reduced, and was essentially no significant difference from that of the DiD NPs treated group (Fig. 3E–G). The above results suggested that MM@DiD NPs had great potential to enhance uptake of inflammatory macrophages by the active target effects. Thus, it was proved that MM-camouflaged biomimetic NPs have a specific target ability based on the CCL2-CCR2 axis against atherosclerotic plaques.

AS was an inflammatory disease, in which circulating oxidized lipids activate endothelial cells, leading to the monocytes being recruited and differentiated into lipid-enriched macrophages (foam cells) and the subsequent formation of lesions in the arterial wall [43]. CCL2 secretion by endothelial cells, macrophages, etc. in atherosclerotic lesions is enhanced, anchored to the plasma membrane of endothelial cells by glycosaminoglycans, and bound to CCR2 from circulating monocytes, facilitating their adhesion to endothelial cells and migration to the subendothelial space [31, 44–46]. Owing to MM@MTX NPs remaining CCR2 protein of MM on the surface, MM@MTX NPs could be specifically delivered to the inflammation macrophage by the CCL2-CCR2 axis [47]. It suggested that MM@MTX NPs could provide an effective strategy for targeting inflammatory macrophages.

### In vitro biocompatibility assessment

The cell compatibility of nanomedicines is an essential requirement for further applications. We evaluated the safety of three MTX formulations on HUVEC cells (ECs), RAW 264.7 cells and LPS-induced RAW 264.7 cells (Additional file 1: Fig. S8). At a relatively low concentration of MTX (≤ 2.5 μg/mL), three MTX formulations showed no significant cytotoxicity to both cell lines. However, the cytotoxicity of the free MTX and the MTX NPs was gradually increased with the increased concentration. Both MTX NPs and MM@MTX NPs treated groups showed a low cytotoxicity to ECs at all the tested concentrations, and only free MTX showed the significant toxicity at the high concentration (20 μg/mL). Although MM@MTX NPs showed cytotoxicity against RAW 264.7 at high concentration (20 μg/mL) as well as LPS-induced RAW 264.7, the cell survival rates were all significantly higher than those in the free MTX and the MTX NPs groups. These results suggested that MM@MTX NPs have a favorable safety profile to cells for significantly ameliorating the side effects of MTX for further applications.

Nanomedicines might cause adverse effects, such as hemolysis of normal red blood cells after intravenous administration, leading to circulatory dysfunction [48], especially for cyclodextrin-based biomaterials with a potentially high risk of hemolysis. As shown in Additional file 1: Fig. S9, the hemolysis of three MTX formulations was lower than 5% at all investigated concentrations, which suggested that the NPs met the hemolysis requirement of biomaterials [49]. The images showed no hemolysis at all concentrations of free MTX, MTX NPs, or MM@MTX NPs, compared with the positive control (Additional file 1: Fig. S9). The hemolytic results revealed that three formulations of MTX had favorable hemocompatibility.

Further zebrafish embryo experiments were used to verify the developmental toxicity. In Additional file 1: Fig. S10, most groups of zebrafish embryos had a 100% hatching rate and no serious deformities in the zebrafish larvae. Only the free MTX group showed one developmentally malformed zebrafish embryo at 72 h. These results indicated that MTX NPs and MM@MTX NPs were biocompatible and safe for potential applications in vivo.

MTX was a commonly used anti-tumor and immunomodulatory compound that had gained widespread acceptance in the administration of rheumatoid arthritis, psoriasis, nodular disease and many oncological conditions [50]. Although MTX was generally considered safe and easy to use, it was associated with some adverse effects. When MTX was encapsulated into NPs and subsequently coated by MM, the biocompatibility of MTX was significantly improved, resulting from the polymeric carrier and MM protected cell from direct exposure to MTX. These results confirmed that the biomimetic nanocarriers might be a well-rounded candidate for drug delivery with low cytotoxicity.

### In vitro anti-atherosclerotic effects

**Fig. 4:**
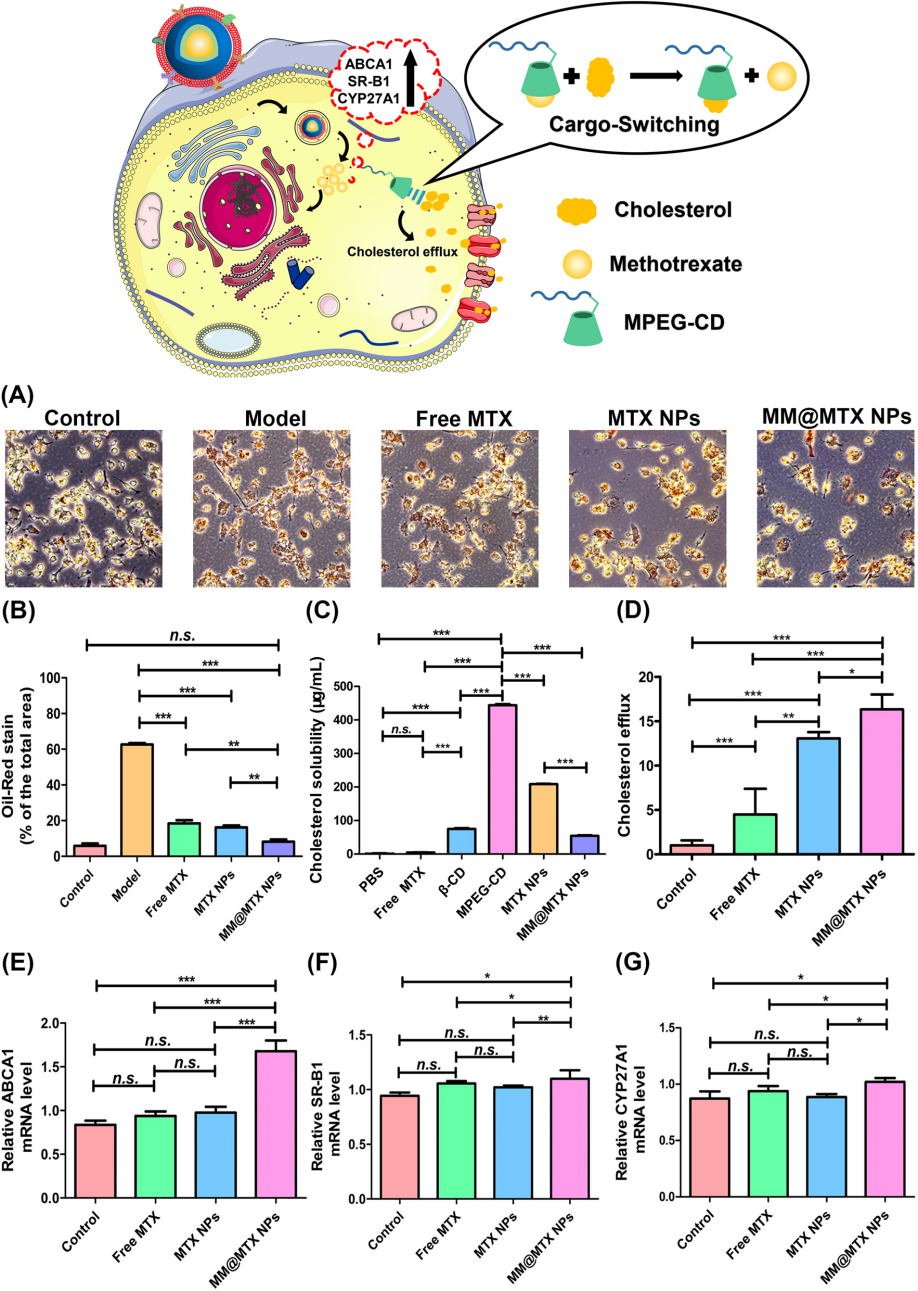
**A** Optical microscope images and **B** quantitative analysis of the oxLDL-induced foam cells in RAW 264.7 cells after treatment with free MTX, MTX NPs, MM@MTX NPs (equal to MTX with 1 µg/mL). (Bar = 40 µm). **C** Cholesterol efflux from RAW264.7 cells treated with different MTX formulations. The dose of free MTX was 1 µg/mL. **D** CCs solubilization of PBS, free MTX, β-CD, MPEG-CD, MTX NPs and MM@MTX NPs at physical condition. Quantification of **E** ABCA1, **F** SR-B1 and **G** CYP27A1 mRNA levels in RAW 264.7 cells by real-time PCR. (*n* = 5, *p < 0.05, **p < 0.01, ***p < 0.001; *n.s.,* no significance)

Lipid metabolism of macrophages is closely related to the degree of macrophage foam cell formation and the severity of atherosclerosis [51]. Studies have shown that expression of ABCA1, SR-B1 and CYP27A1 were increased could promote cholesterol efflux, which was considered an important strategy for inhibiting the progression of atherosclerosis [11, 52, 53]. From the result of the oxLDL-induced foam cell investigation, MTX and MTX NPs could significantly enhance the lipid efflux from macrophages to inhibit the formation of foam cells (Fig. 4A, B). MTX could significantly enhance cholesterol efflux, compared with the model group, MTX NPs and MM@MTX NPs exerted enhanced cholesterol efflux-promoting activity (Fig. 4C). It was worth noting that MM@MTX NPs showed a relatively stronger efficacy to inhibit the foam cells form compared to equal drug concentration treatment of MTX and MTX NPs, which should owe to the synergetic effects for the enhanced solubility of CCs by MPEGylated β-CD (Fig. 4D) and the enhanced uptake of inflammatory macrophages mediated by the biological activity of MM (discussed in the section of *Characterization of the targeted delivery *in vitro). These showed MM@MTX NPs could inhibit foam cells' form by significantly increasing the cholesterol efflux and the CCs solubility, which was extremely important for inhibiting the progression of AS.

To further investigate the cholesterol efflux effect of MM@MTX NPs. The mRNA expression of ABCA1, SR-B1 and CYP27A1 was quantified in RAW 264.7 after treatment through the different MTX formulations. Although free MTX and MTX NPs treated groups could slightly increase the mRNA expression of ABCA1, SR-B1 and CYP27A1 with no significant difference, the mRNA expression of ABCA1, SR-B1 and CYP27A1 was a significant difference for MM@MTX NPs treated group (Fig. 4E–G). Notably, MM@MTX NPs had a much more significant effect on the mRNA expression upregulation of ABC1 than that of SR-B1 and CYP27A1. These results revealed that the main mechanism for MM@MTX NPs promoted cholesterol efflux was contributed to the local MTX delivery for enhancing the reverse cholesterol transport through up-regulating the biological functions of ABCA1, SR-B1 and CYP27A1. Meanwhile, the hydrophobic cavity of β-CD was able to dissolve the CCs and subsequently accelerate the cholesterol efflux (Fig. 4D) [54]. These prove that MM@MTX NPs can inhibit the foam cells formation by promoting cholesterol efflux.

As previous reports, the accumulation of CCs will further exacerbate inflammation [6–8]. Therefore, cholesterol clearance will be beneficial to regulate the pathological inflammation. On this note, mRNA expression of TNF-α, IL-1β and interferon-β (IFN-β) was quantified in RAW 264.7 after treatment by the different MTX formulations. From the results (Additional file 1: Fig. S11), the free MTX, MTX NPs and MM@MTX NPs treatment groups were able to effectively inhibit the mRNA expression of TNF-α and IL-1β, compared with the control group. In particular, the MM@MTX NPs treated group had a much more significant reducing effects on the mRNA expression of TNF-α, IFN-β and IL-1β than that of the other treated groups as free MTX, MTX NPs, which should be resulted from the enhanced uptake of MM@MTX NPs by the inflammatory cells and the efficient clearance of CCs through the reverse cholesterol transport. Therefore, MM@MTX NPs with the enhanced cellular uptake and the cholesterol-promoting efflux properties had been confirmed to not only inhibit the formation of foam cells, but also alleviate the inflammation by removing the overloaded CCs through reverse cholesterol transport.

### In vivo atherosclerotic plaque targeting and long-term circulation

**Fig. 5:**
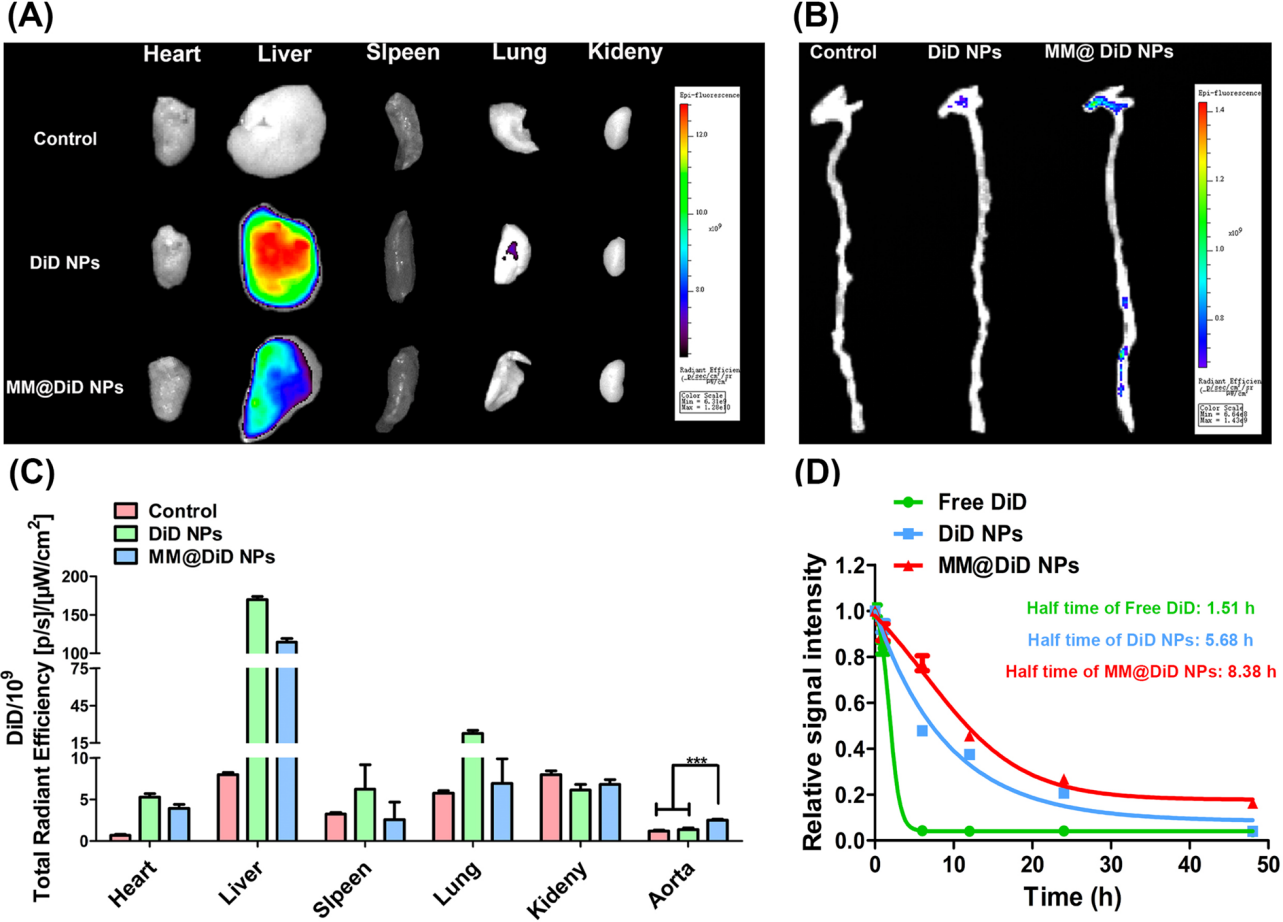
Representative fluorescence images of DiD fluorescent signal accumulated in the **A** main organ and **B** aorta after 24 h post-injection. **C** Quantitative of DiD fluorescence intensity in main organ and aorta. **D** Relative fluorescence intensity of free DiD, DiD NPs, and MM@DiD NPs in blood. (*n* = 3, ***p < 0.001)

AS plaque lesion was started with the damaged endothelial cells of the blood vessels. Macrophages and smooth muscle cells deposit cholesterol in vessel wall to form pathological foam cells, ultimately leading to the thickening of the arterial blood vessel walls as plaques. During the above progression of AS plaque, the increasing CCL2 secreted in macrophages and endothelial cells would further recruit much more macrophages accumulating in lesions [31]. Therefore, MM@DiD NPs integrated with the homing activity were expected to not only escape immune clearance but also enhance targeting cargo delivery to the AS plaque. To accurately monitor the plaque targeting ability and distribution of the NPs, fluorescence imaging of major organs and aorta was performed 24 h after injection of DiD NPs and MM@DiD NPs (Fig. 5A, B). Compared with DiD NPs group having a strong fluorescence in the liver and little fluorescence in the lung and aorta, MM@DiD NPs could significantly reduce the undesirable delivery and accumulation into the liver. Meanwhile, for AS aorta, the fluorescence of the MM@DiD NPs treated group was significantly stronger, approximately two times than that of DiD NPs treated group (Fig. 5C).

To further assess whether MM@DiD NPs inherit the long-circulating lifespan of natural MM, pharmacokinetics was investigated in C57BL/6. Interestingly, free DiD almost disappeared from the blood at 6 h after injection, but 47.77% of DiD NPs and 77.15% of MM@DiD NPs in the blood at the same time (Fig. 5D). The half-time of free DiD, DiD NPs and MM@DiD NPs was 1.51, 5.68 and 8.38 h, respectively. These results show that MM@DiD NPs can significantly extend the blood retention time, thereby enhancing the targeted efficiency of AS.

### In vivo anti-atherosclerosis efficacy

**Fig. 6:**
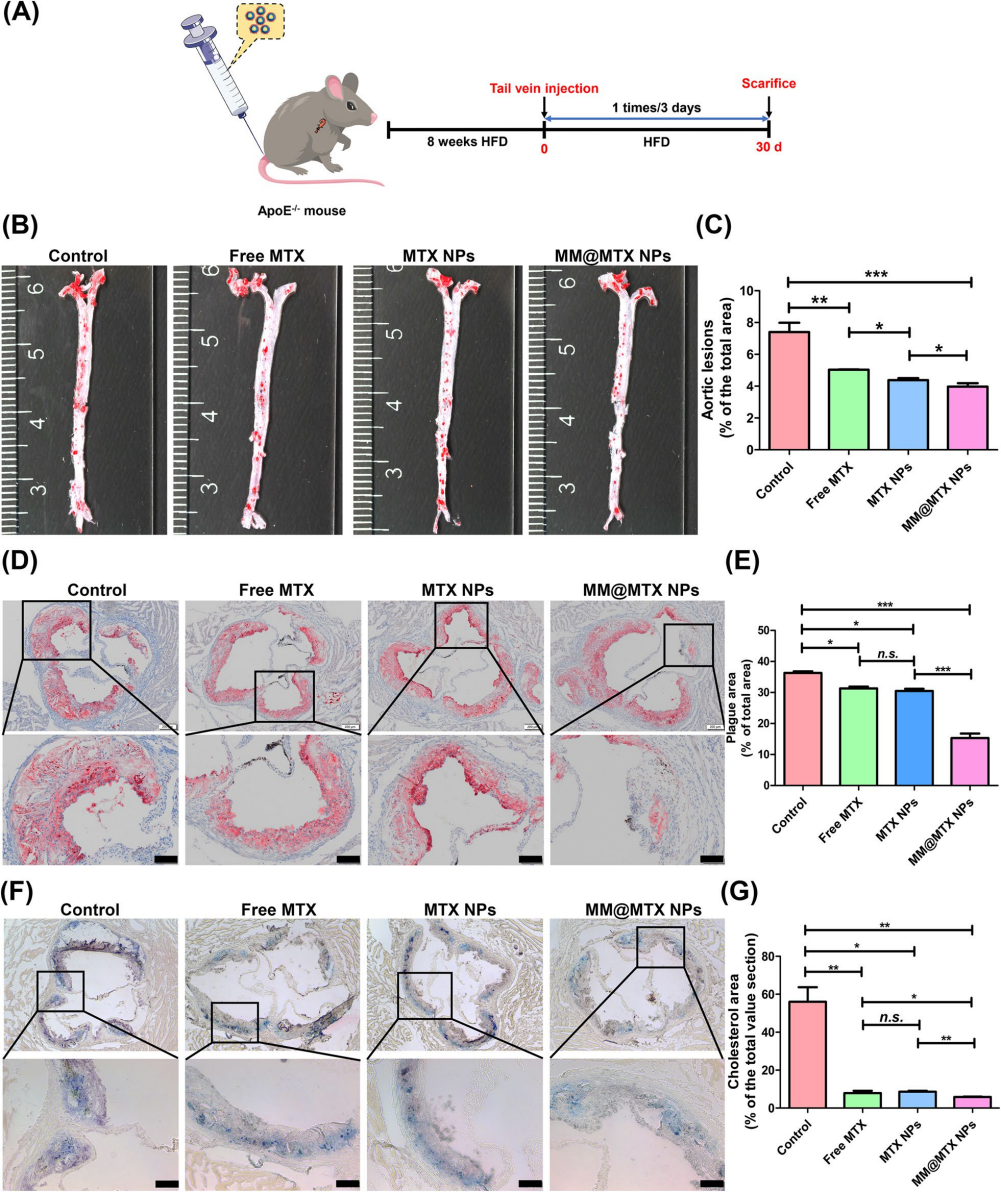
**A** A schematic diagram of the experimental design. **B** A representative photomicrograph of the aorta with frontal ORO staining. **C** The quantification assessment regarding the lesion area (*n* = 5). **D** Cross-section of ORO-stained aortic root (Bar = 100 μm). **E** The quantification of the area of lipid deposition on cross sections in the aortic root (*n* = 5). **F** Cholesterol areas with Schultz method staining (Bar = 100 µm). **G** The area of cholesterol deposition on cross-sections of the aortic root was quantified (*n* = 5). (*p < 0.05, **p < 0.01, ***p < 0.001 and *n.s.*, no significance)

Anti-atherosclerotic effects were further investigated in ApoE^−/−^ mice as a pathological model of AS. 30 days after treatment, the aortas were dissociated and dyed with ORO (Fig. 6A). Frontal photomicrographs of an ORO-stained aorta reveal that the treatment with MM@MTX NPs effectively depressed the development of AS (Fig. 6B). To quantitatively assess AS lesions, the area ratio of the lesion to the aorta was calculated. In Fig. 6C, compared with the control group, free MTX and MTX NPs treated groups had a significant therapeutic effect and MM@MTX NPs treated group showed a much more pronounced therapeutic effect. Although MTX was an anti-inflammatory agent can alleviate the progression of AS by upregulating ABCA1, SR-B1 and CYP27A1 gene expression to induce cholesterol reversal transport. However, the poor solubility would result in low bioavailability when administered via tail vein. The plaque ratios of AS lesions were 5.0% and 4.3% in the free MTX and MTX NPs treatment groups, respectively. Notable, the plaque ratio of AS lesion was significantly low to 3.8% in MM@MTX NPs (Fig. 6C). To further investigate the formation of deposition of lipids in AS plaques, ORO was used to stain cross-sections of aortic roots, respectively. According to ORO-stained, plenty of lipids were deposited in the control plaques, up to 36.40%, and the lipid deposition ratios were reduced to 30.10% and 31.10% in the free MTX and MTX NPs treated groups, respectively (Fig. 6D, E). Notably, among them, MM@MTX NPs treated group exhibited a significant reduction to 15.41% of the lipid deposition in the plaques (Fig. 6D, E).

Previous studies have reported that MTX and β-CD could promote cholesterol efflux to therapy AS [11, 54]. Here, the content of cholesterol was detected in the aorta by the Schultz method, in which cholesterol or cholesterol ester would be stained green or purple (Fig. 6F). Images analysis for cholesterol showed that the control group had a severe cholesterol deposit in the aorta, up to 56.0% (Fig. 6G). The deposition cholesterol in MTX and MTX NPs treated groups significantly decreased to 7.88% and 8.65%, respectively. Especially, after treatment with MM@MTX NPs, the level of cholesterol was significantly reduced to about 5.0% in the AS plaques. All of these findings demonstrate that MM@MTX NPs can notably suppress cholesterol deposition and thus significantly delay AS plaque progression.

**Fig. 7:**
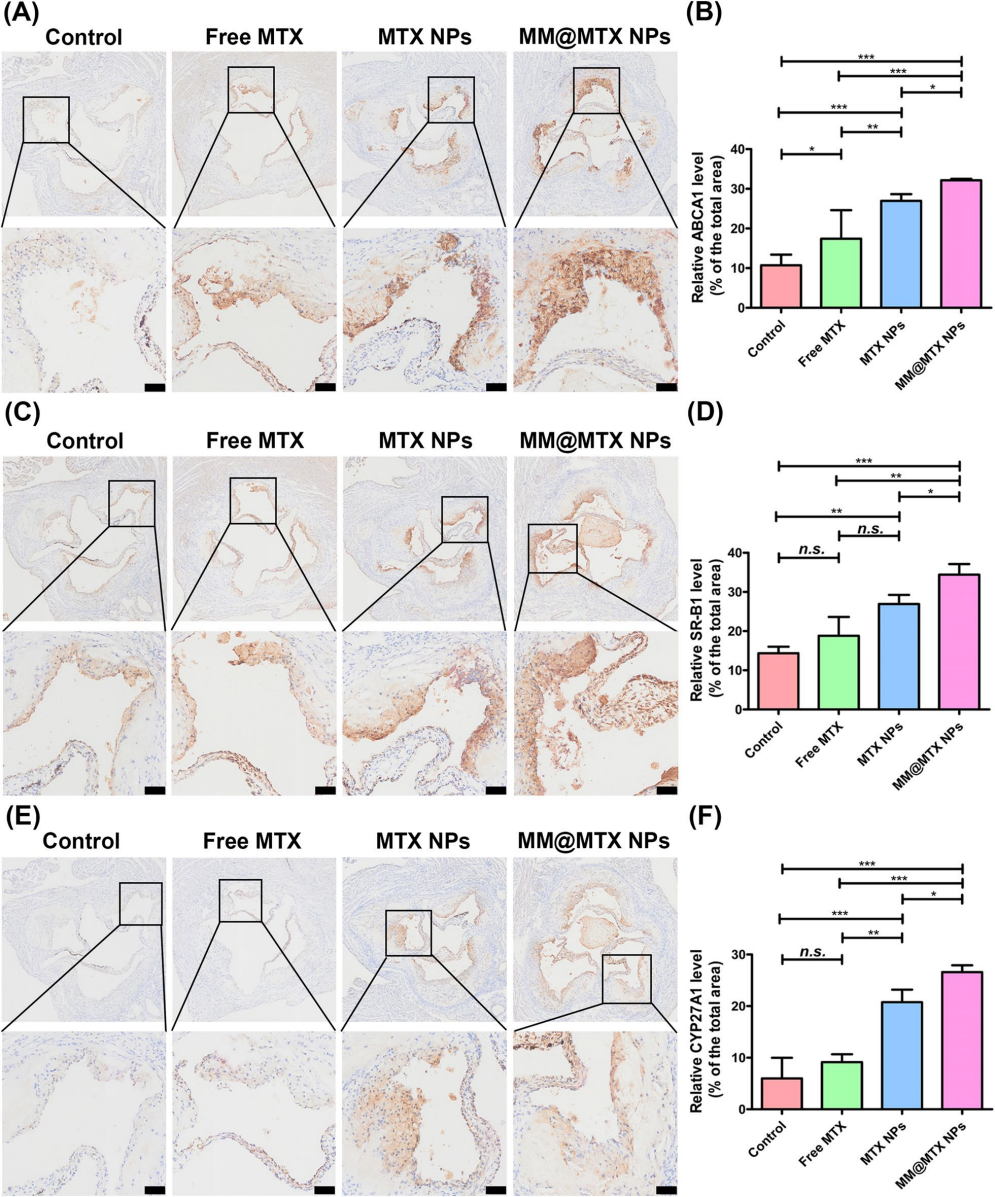
Photographs of representative immunohistochemical staining and quantification analysis of (**A**, **B**) ABCA1, **C**, **D** SR-B1 and **E**, **F** CYP27A1 (*n* = 5). (Bar = 100 µm). (*p < 0.05, **p < 0.01, ***p < 0.001, *n.s.*, no significance.)

As the previous reports, MTX has the activities to promote the efflux of CCs by up-regulating the expression of SR-B1, ABCA1 and CYP27A1 [11, 13]. Immunohistochemical analysis of ABCA1, SR-B1 and CYP27A1 showed that the MM@MTX NPs treated group was able to effectively elevate the expression levels of ABCA1, SR-B1 and CYP27A1 in the plaque lesion, which could be beneficial to promote the overloading of CCs efflux in the atherosclerotic lesion and subsequently delay the progression of AS (Fig. 7).

**Fig. 8:**
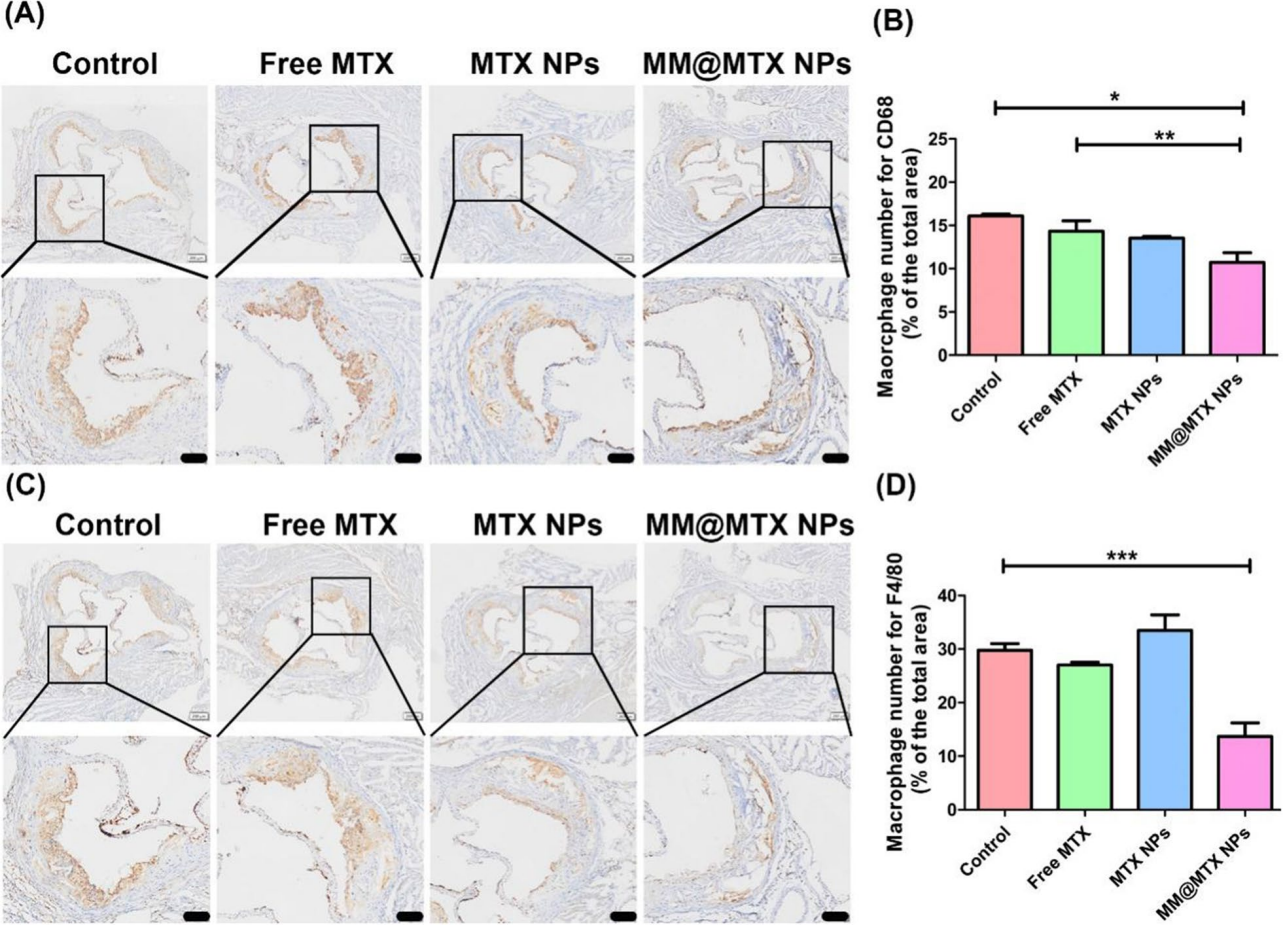
Photographs of representative immunohistochemical staining and quantification analysis of **A**, **B** CD68 and **C**, **D** F4/80 (*n* = 5). (Bar = 100 µm). (*p < 0.05, **p < 0.01, ***p < 0.001.)

Numerous studies have reported that aberrant macrophage proliferation promotes the progression of AS [32]. Immunohistochemical analysis of CD68 (Fig. 8A, B) and F4/80 (Fig. 8C, D) as macrophage markers demonstrated that the content of macrophages was significantly low in the MM@MTX NPs group, which confirmed that MM@MTX NPs could significantly alleviate the abnormal macrophages in the atherosclerotic lesion, thereby delaying the progression of AS. Overall, MM@MTX NPs could be exploited as advanced nanocarriers with the long-term circulation and active targeting of inflammatory macrophage foam cells for the target delivery into the atherosclerotic lesion. In addition, the target local cargo release and the β-CD enhanced solubility of cholesterol crystal could significantly promote cholesterol efflux from foam cells as well as alleviate inflammatory response in the lesion and ultimately inhibit AS progress.

**Fig. 9:**
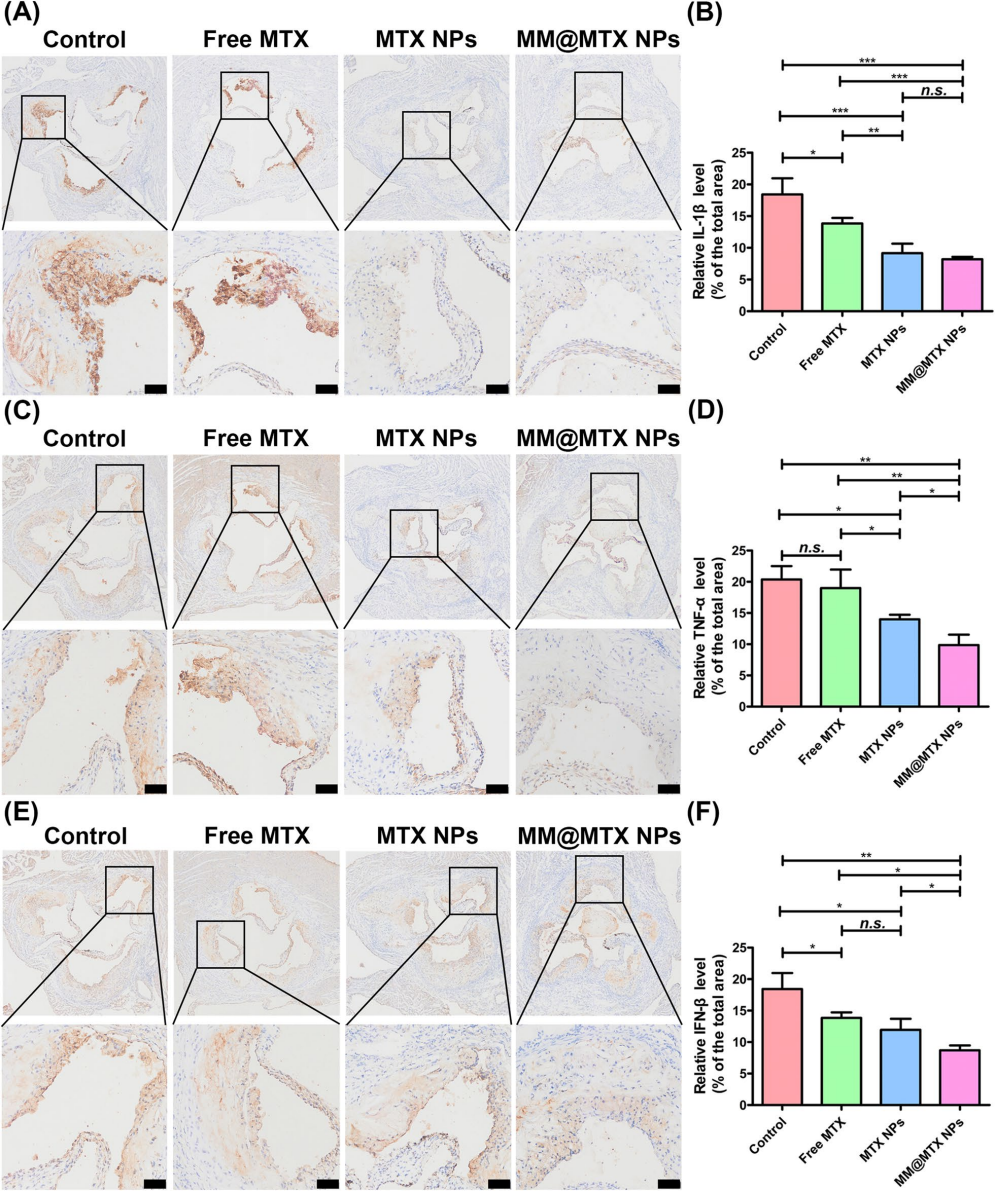
Photographs of representative immunohistochemical staining and quantification analysis of **A**, **B** IL-1β, **C**, **D** TNF-α and **E**, **F** IFN-β (*n* = 5). (Bar = 100 µm). (*p < 0.05, **p < 0.01, ***p < 0.001, *n.s.*, no significance.)

Overloaded CCs always lead to induce the aberrant inflammatory cytokine secretion, which results in the undesirable inflammation. Inflammations have a key role in the development of atherosclerosis, favoring the continued recruitment of circulating monocytes within the vascular lesion and supporting their maturation in macrophages and eventually foam cells [55]. Thus, effective control of inflammation should also be an important strategy in assessing the effectiveness of treatment. As shown in Fig. 9, compared with the other treated groups MM@MTX NPs showed superior in reducing expression of the inflammatory factors in the plaque lesion, such as IL-1β, TNF-α, and IFN-β. Therefore, benefiting from the targeted drug delivery and the multipotency CCs reverse efflux and ant anti-inflammatory activities of MTX, MM@MTX NPs was confirmed to be efficient for atherosclerosis management.

### Blood evaluation

**Fig. 10:**
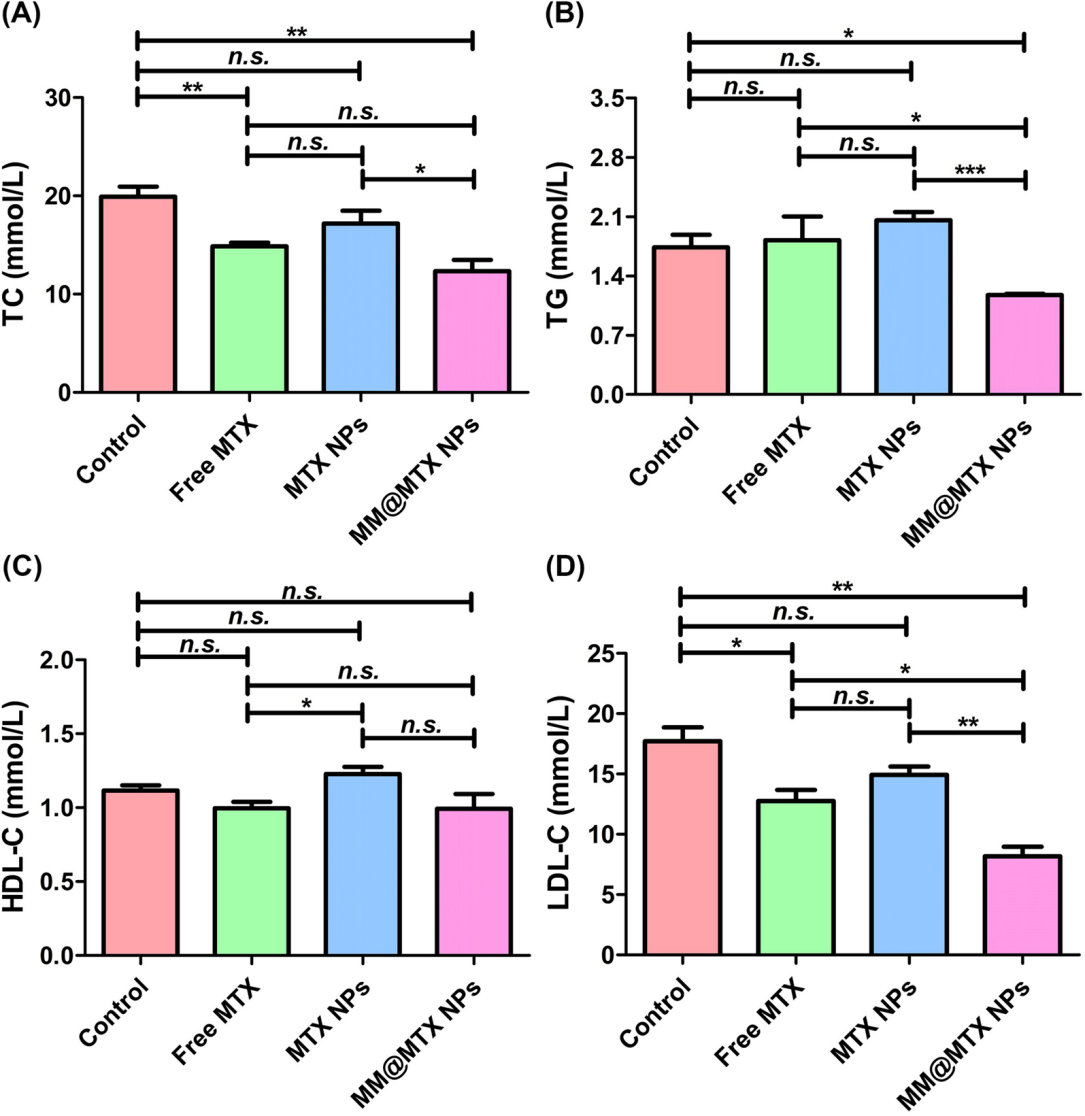
Lipid content parameters of **A** TC, **B** TG, **C** LDL-C and **D** HDL-C in serum. (*n* = 5, *p < 0.05, **p < 0.01, ***p < 0.001 and *n.s.*, no significance.)

Sterols could be incorporated into the hydrophobic cavities of β-cyclodextrin, resulting in stable inclusion complexes. Therefore, β-cyclodextrins exhibiting a high complex-forming ability with lipids were able to use as an effective regulator of cholesterol metabolism in vivo. It has been shown that β-cyclodextrin can reduce plasma triglyceride and cholesterol levels [10]. Herein, the serum samples were analyzed (Fig. 10A–D). According to the serum analysis, among all treated groups, the contents of total cholesterol (TC), low-density lipoprotein cholesterol (LDL-C) and triglycerides (TG) for MM@MTX NPs treated group were significantly lower, and high-density lipoprotein cholesterol (LDL-C) was no significance to control. These results implied that MM@MTX NPs inherited functions of the original β-CD to reduce the contents of plasma triglyceride and cholesterol for inhibiting AS progression.

### Biosafety assessment

**Fig. 11:**
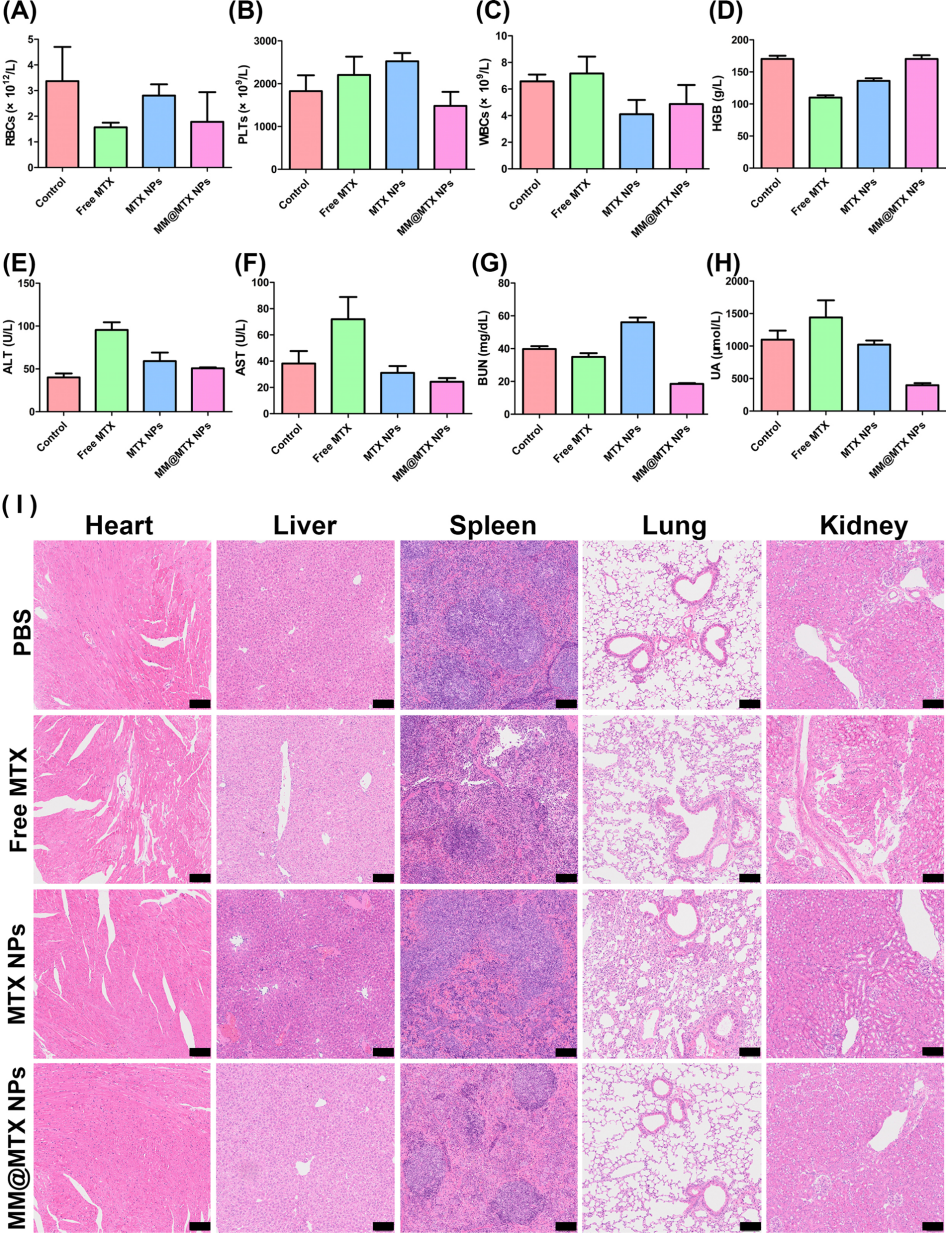
**A**–**D** Bloodological parameters of treatment groups (*n* = 5). **E**–**H** Biochemical functional parameters of liver and kidney (*n* = 5). **I** H&E-stained sections of major organs. (Bar = 100 µm)

The toxic effects of NPs on various organs are an important issue in drug therapy [56]. To evaluate safety, investigation of potential toxicity was performed after treatment. The weight of all mice has no significant change (Additional file 1: Fig. S12). Complete blood counts implied also no significant change (Fig. 11A–D). Specifically, the counts of immune-related cells, in the blood of treated mice were similar to those of the control mice (Fig. 11C, Additional file 1: Fig. S13). Biochemical clinical analysis indicated normal biological function of the liver and kidneys (Fig. 11E–H). The results of H&E staining also showed the biocompatibility of MM@MTX NPs (Fig. 11I). Thus, MM@MTX NPs indicated no significant toxicity after treatment, suggesting that MM@MTX NPs could be a safe candidate for the potential treatment of chronic vascular diseases.

## Conclusion

In summary, we have successfully developed the MM-camouflaged biomimetic NPs (MM@MTX NPs) with favorable long-term circulation and AS plaque targeting capabilities. MM@MTX NPs can enhance the target cargo delivery and sustained local drug release in AS plaques for effectively inhibiting the development of AS, mainly resulting from the promoting reverse cholesterol transport and controlling lipid levels of blood. Additionally, MM@MTX NPs exhibit the desirable safety profile, without the significant side effect in vitro and in vivo. Thus, MM@MTX NPs represent the novel advanced nanocarriers as the effective target drug delivery system for the potential treatment in AS.

## Materials and methods

### Material

Amino-Methoxy polyethylene glycol (NH_2_-MPEG, MW = 5000 Da) was purchased from Ponsure Biological CO., Ltd (Shanghai, China).β-CD, *p*-toluene sulfonyl (PTS), Oil Red O (ORO) and cholesterol were obtained from Shanghai Aladdin Biochemical Technology Co., Ltd. (Shanghai, China). Methotrexate was purchased from Meilun Co., Ltd (Dalian, China). 1, 19-Dioctadecyl-3, 3, 39, 39-tetramethylindodicarbocyanine perchlorate (DiD) was supplied by Biotium Inc (Fremont, US). Bindarit was obtained form Macklin Inc (Shanghai, China). Lipopolysaccharide (LPS) was purchased from Solarbio (Beijing, China). Human high-oxidized low-density lipoprotein (ox-LDL) and high-density lipoprotein (HDL) were obtained from Yiyuanbiotech (Guangzhou, China). MTS was obtained from Abcam Co., Ltd (Shanghai, China). Dulbecco’s Modified Eagle’s Medium (DMEM) and Gibco RPMI 1640 Medium were bought from Hyclone Co., Ltd. Total Cholesterol (TC) Quantitation Kit was purchased from Elabscience Co., Ltd (Wuhan, China). Other reagents were analytical grades.

### Preparation of MPEG-CD

The β-CD group was functionalized by NH_2_-MPEG for MPEG-β-CD according to the reported method [57]. The synthetic procedure of MPEG-CD consists of two steps from β-CD which course of the reaction was shown in Additional file 1: Fig. S1.

#### Synthesis of β-CD-ptoluenesulfonyl (CD-OTS)

Briefly, 5.0 g of β-CD was dissolved in a 1% NaOH solution. Acetonitrile solution containing 0.85 g of p-TsCl in 2.5 mL was added to the above solution and reacted for 6 h at room temperature. Then, the pH was adjusted to ~ pH 7 with hydrochloric acid solution (1 mM). The crude product was obtained by filtration, washed three times with ethanol, and dried in vacuum at 40 °C for 24 h to obtain a pure white solid.

#### Synthesis of MPEG-CD

Next, 0.5 g NH_2_-MPEG and 0.15 g CD-OTS were added to dimethyl sulfoxide (DMSO). After stirring at room temperature for 24 h, the reaction solution was dialysis in water and freeze-drying to obtain MPEG-CD.

### Preparation of the macrophage membranes

Macrophage membranes were isolated and obtained refer to previously reported [22]. Briefly, cells were collected and added to the membrane protein extract and incubated in an ice bath for 15 min. The cell suspension was transferred to a homogenizer and homogenized about 30 times. The mixture was centrifuged (4 °C, 14,000 rpm, 30 min) to obtain cell membranes. MM vesicles were obtained by sonicating the macrophages for 15 min and the Avastin mini extruder (Avastin, LF-1, Canada) squeezed the macrophages through a 400 nm polycarbonate porous membrane 10 times. MM vesicles were stored in water at 4 °C.

### Preparation of MPEG-CD@MTX (MTX NPs)

MTX-loaded NPs (MTX NPs) were prepared by a modified self-assembly method. In brief, MPEG-CD (75 mg) and MTX (5 mg) were dissolved in DMSO (1 mL) and then dialyzed against distilled water. The solution was filtered by 0.22 µm microporous membrane to remove the unloaded drug. The MTX NPs solution was stored at 4 ℃. The content of MTX was determined by UV–Vis Spectrophotometer (Beckman Coulter, DU730, USA) at the wavelength of 300 nm.

### Preparation of MM encapsulated MTX NPs (MM@MTX NPs)

MM@MTX NPs were made by encapsulating MM on the surface of MTX NPs by direct extrusion. Briefly, MM vesicles and MTX NPs were mixed in a specific ratio and sonicated (FS30D, 42 kHz, 100 W) for 3 min. The mixture was then extruded 10 times through a 400 nm polycarbonate porous membrane using an Avestin mini extruder (Avestin, LF-1, Canada) to harvest the MM@MTX NPs solution. The drug loading (DL%) and encapsulation efficiency (EE%) of NPs were calculated according to the previous reported [58].

### Characterization of MTX NPs and MM@MTX NPs

FT-IR spectra of free MTX, blank NPs and MTX NPs were recorded by KBr method on FT-IR spectrometer. X-ray diffractometer was used to measure the X-ray diffraction of free MTX, blank NPs and MTX NPs by Cu Kα radiation in the range of 3–50°. MTX, MPEG-CD and MPEG-CD@MTX powder samples were diluted to ~ 10 mg/mL solution in deuterated dimethyl sulfoxide-d6 (DMSO-d6) for ^1^H-NMR spectra and 2D NOESY spectra of analysis.

The particle size and zeta potential of MTX NPs and MM@MTX NPs were measured with Malvern Zetasizer ZS90 (Malvern, Nano ZS 90, U.K.). The morphologies of the MTX NPs and MM@MTX NPs were observed with transmission electron microscopy (TEM) at 200 kV (JEOL, JEM-2100F, Japan).

The stability of MM@MTX NPs was monitored by measuring size and PDI in PBS solution (10 mM, pH 7.4, 37 ℃) which contained 5% FBS at the predetermined time interval.

### Identification of the membrane proteins for MM@MTX NPs

Membrane proteins were characterised by polyacrylamide gel electrophoresis (SDS-PAGE). Collected membrane proteins received using Bio-Rad electrophoresis system were run on 4- to 12% Bis–Tris 10-well mini-gels at 75 V for 0.5 h, followed by 140 V for 1 h. Finally, visualization of the resulting polyacrylamide gels was carried out by overnight staining with SimplyBlue.

The amount of CCR2 and CD47 in the samples was determined by Western blot analysis. Total MM protein extracted from 1 × 10^7^ RAW 264.7 cells and subsequently MM@MTX NPs, was extracted using the Total Cell Protein Extraction Kit. Samples were electrophoresed on 10% SDS–polyacrylamide gels and transferred to polyvinylidene difluoride membranes (Millipore, USA). Membranes were then treated with primary anti-CCR2 (Abcam, anti-CCR2 antibody ab203128, USA) and CD47 (Abcam, anti-CD47 antibody ab175388, USA) followed by horseradish peroxidase-labelled goat/anti-rabbit IgG (H + L) (Beyotime, Jiangsu, China). Protein signals were measured by enhanced chemiluminescence and visualized with a ChemiDoc MP imaging system (Bio-Rad, ChemiDoc MP, USA).

### In vitro drug release study

The drug release from MTX NPs and MM@MTX NPs was studied with the dialysis method. 1.0 mL of MTX NPs and MM@MTX NPs (equivalent to MTX 400 μg/mL) solution were transferred into a dialysis bag (MW = 3500 Da) and immersed into 49 mL of PBS (containing 5% (V/V) tween-20) with 20 mM cholesterol or without cholesterol. The release system was continuously vibration (100 rpm) at 37 ℃. At the specified time, 1 mL of release solution was taken out for the measurement and 1 mL of fresh release medium was replaced. The content of released MTX was measured by UV–Vis Spectrophotometer (Beckman Coulter, DU730, USA) at the wavelength of 300 nm. The MTX accumulative release percentage was calculated and described as the mean ± standard deviation (SD) of the five replicates, and its accumulative release curve was plotted.

### In vitro cellular uptake

AS is a chronic inflammatory disease, therefore LPS-induced inflammatory macrophages were chosen as an in vitro model. RAW 264.7 cells were seeded at a density of 5 × 10^5^ cells/well in 6 wells and cultured overnight at 37 °C (5% CO_2_). After that, RAW 264.7 cells were stimulated with 100 ng/mL LPS for 24 h to obtain inflammatory RAW 264.7 cells. MM@DiD NPs or DiD NPs solutions were then added to each well and co-cultured for 1 or 3 h, respectively. Subsequently, cells were washed 3 times with PBS and fixed with paraformaldehyde (PFA) for 15 min and stained with DAPI. The cells were washed 3 more times with PBS and observed with a fluorescent inverted microscope (Thermo Fisher Scientific, EVOS M7000, USA). The fluorescence intensity of the cell absorption images was quantified using Image J software (National Institutes of Health, Image J 2.1, USA). On the other hand, after 1 or 3 h of treatment with samples, cells were washed, digested and collected for analysis on a flow cytometer (Becton Dickinson, Influx, USA) and data were analysed using FlowJo (Tree Star, FlowJo 10.8, USA). To clarify that the increased inflammatory cellular uptake of MM@MTX NPs during targeted delivery in vitro is due to the CCL2-CCR2 that MM coatings possess. Here, inflammatory RAW 264.7 cells CCL2 inhibition was carried out by bindarit (5 μg/mL) [59], and the experimental steps above were subsequently repeated.

### In vitro cytotoxicity

#### In vitro cell cytotoxicity evaluation

ECs, RAW 264.7 and LPS-induced RAW 264.7 cells were seeded at a density of 1.0 × 10^4^ cells per well in 96-well plates overnight. They were co-incubated with media containing different doses of free MTX, MTX NPs and MM@MTX NPs. After 24 h of incubation, cell viability was quantified using the MTS assay.

#### In vitro hemocompatibility testing

As previously reported [60], hemolysis of MM@MTX NPs was tested by in vitro direct contact method. Briefly, 1 mL of rabbit blood was diluted with 1.25 mL of saline. Then, 0.1 mL of the diluted whole blood sample was added to different concentrations of free MTX, MTX NPs or MM@MTX NPs solutions (1.25 to 20 µg/mL). The samples were then incubated at 37 °C for 1 h. Subsequently, the solutions were centrifuged at 3000 rpm for 5 min. The absorbance of the supernatant was measured at 540 nm using a microplate reader (Bio-Tek, μQuant, USA). Distilled water and saline were used as positive and negative controls, respectively.

#### In vivo developmental toxicity in zebrafish

Zebrafish AB strains were used for in vivo developmental toxicity experiments. Free MTX, MTX NPs and MM@MTX NPs were mixed with fish water to form different concentrations of MTX solutions (5, 10 to 20 µg/mL). These solutions were then incubated with healthy AB wild type zebrafish embryos (12 h after fertilisation) and replaced daily with fresh medium. Embryo survival, larval growth and malformation were observed at 24, 48 and 72 h using a stereomicroscope (Zeiss, SteREO Discovery V20, Germany).

### Foam cell formation and cholesterol quantification

Reference to previous reports and refinements to establish foam cells [12, 57]. RAW 264.7 cells were inoculated in 6-well plates at a cell density of 2 × 10^5^ per well, cells were then stimulated in medium containing 100 ng/mL LPS with 50 μg/mL ox-LDL to form foam cells. Cells were treated with free MTX, MTX NPs or MM@MTX NPs (equivalent to 0.5 μg/mL MTX) for 24 h. The medium was removed, washed twice with PBS and fixed in 4.0% PFA solution. Oil Red O (ORO) working solution was stained for 15 min. Cells were observed with a microscope (Leica, DMI1, Japan) and images were obtained. Quantitative red staining was performed using ImageJ software.

### In vitro cholesterol efflux assay

In vitro cholesterol efflux was performed using RAW 264.7 macrophages as previously reported [57]. After RAW 264.7 cells were treated with free MTX, MTX NPs or MM@MTX NPs (equivalent to 1 μg/mL MTX), the medium was replaced with medium containing cholesterol (5 μM) for 4 h. Cells were washed twice, and replaced with 25 μg/mL HDL medium. After 4 h, collect aliquots of supernatant. Cells were lysed with 0.1% Triton-X100. The cholesterol content of the medium and cell lysate was measured using a Total Cholesterol Quantification Kit. The results are described as a ratio of the cholesterol content in the medium to the cholesterol content in the cell lysate.

### CCs solubilization assay

CCs were incubated with free MTX, β-CD, MPEG-CD, MTX NPs or MM@MTX NPs at PBS for 12 h. The supernatant was collected after incubation and filtered through a filter membrane (0.8 μm). Filter solution was incubated with ethanol (1:9, V/V) for 30 min under ultrasound and then centrifuged at 12,000 rpm for 5 min. The cholesterol concentration in the supernatant was measured using a Total Cholesterol Quantification Kit.

### Gene expression

The gene expression of CYP27A1, ABCA1, SR-B1, IL-1β, TNF-α and INF-β were assessed. RAW 264.7 cells were seeded into 6-well plates at a density of 4 × 10^5^ cells per well. Cells were treated with 50 μg/mL oxLDL (containing 100 ng/mL LPS) for 12 h. Cells were treated with free MTX, MTX NPs and MM@MTX NPs (1.0 μg/mL of MTX) for 24 h. RNA was extracted by RNAeasy Plus Mini Kit (Qiagen, RNAeasy Plus Mini Kit, Germany) and quantified by NanoDrop2000 (Thermo Scientific, NanoDrop2000, USA). The β-Actin gene expression as a housekeeping gene.

Primer pair sequences as following: ABCA1: 5′-ACAGTGGCGGCAACAAACG-3′ and 5′-GCTTAGGGCACAATTCCACAAGA-3′; SR-B1: 5′-CATCTTCTCTACCACCTTGCCT-3′ and 5′-TCAGGCAGCCAATCCTTTTC-3′; CYP27A1: 5′-ACAGTGGCGGCAACAAACG-3′ and 5′-GCTTAGGGCACAATTCCACAAGA-3′; IL-1β: 5′-AACCTGCTGGTGTGTGACGTTC-3′ and 5′-CAGCACGAGGCTTTTTTGTTGT-3′; TNF-α: 5′-GGTGCCTATGTCTCAGCCTCTT-3′ and 5′-GCCATAGAACTGATGAGAGGGAG-3′; INF-β: 5′-GCCTTTGCCATCCAAGAGATGC-3′ and 5′-ACACTGTCTGCTGGTGGAGTTC-3; β-Actin: 5′-GTGAAGGTGACAGCAGTCGGTT-3′ and 5′-GAAGTGGGGTGGCTTTTAGGA-3′.

### In vivo atherosclerotic plaques targeting and long-term circulation test

ApoE^−/−^ mice were fed a high-fat diet (HFD) for 2 months. DiD NPs and MM@DiD NPs were administered via the tail vein at DiD dose of 2 mg/kg. After 24 h, the mice were euthanized and perfused with precooled PBS containing 4% PFA. Each aorta and the main organs were isolated for imaging using a Xenogen IVIS 200 system (Xenogen, IVIS 200, USA).

C57BL/6 mice weighing 25 ± 2 g were subjected to in vivo long-term cycling tests. Briefly, DiD NPs and MM@DiD NPs (200 μL, 2 mg/mL) were injected intravenously and 30 μL of blood was collected rapidly from the tail after 1 min, 1, 6, 12, 24 and 48 h. Blood samples were diluted with 30 μL of PBS containing EDTA-K2 in a 96-well plate and fluorescence intensity was measured using a microplate apparatus (Tecan, M1000 PRO, USA).

### Treatment of atherosclerosis in ApoE^−/−^ mice

ApoE^−/−^ mice after 8 weeks of HFD feeding were randomized into 4 groups (5 mice per group) and dosed for 30 days by tail vein injection every three days. In the treatment groups, mice were administered PBS, free MTX, MTX NPs, or MM@MTX NPs at a dose of 5 mg/kg of MTX.

At the end stage of the treatment, the ApoE^−/−^ mice were euthanized. The pathological evolution was assessed by measuring the lesion area of atherosclerotic plaques in the aorta from the heart to the iliac bifurcation. Briefly, each aorta was fixed with PFA (4% PBS) for 1 h. After washing the peripheral tissue, the aorta was opened longitudinally and the whole aorta was then stained with ORO to quantify the plaque area. The extent of atherosclerotic plaque in the aortic root was determined in the same way. Atherosclerotic plaque areas were quantified using Adobe Photoshop 2021 software.

Aortic root histology and immunohistochemical staining: Aortic roots were fixed in 4% PFA for 1 h. Frozen sections were prepared and stained with toluidine blue to quantify the necrotic core. For analysis of cholesterol and cholesteryl esters, sections were placed in a solution of ferric ammonium sulphate and reacted at room temperature for 3 days. An acetic acid-sulphate mixture was added and subsequently observed under the microscope. For immunohistochemistry, sections were immersed in 3% hydrogen peroxide and 100% methanol for 20 min to inhibit endogenous peroxidase activity, followed by blocking with 1% bovine serum albumin in PBS containing 0.3% Triton X-100 for 60 min. CD68, F4/80, ABCA1, SR-B1, CYP27A1, IL-1β, TNF-α and IFN-β antibodies were co-incubated for macrophage quantification. Histological and immunohistochemical quantification was performed using Adobe Photoshop 2020 software. Hematoxylin–eosin (H&E) staining was used to analyse sections of major organs.

Complete blood routine analysis and serum biochemistry analysis: Blood was collected and analyzed using an automated hematology analyzer (Sysmex Co., Sysmex KX-21, Japan) after treatment.

### Statistical analysis

The data obtained are reported as the mean ± SD in this study. GraphPad Prism Version 7.0 software (GraphPad Software, GraphPad Prism 7.0, USA) was used for the statistical analysis. One-way or two-way analysis of variance (ANOVA) by Tukey’s test was used to reveal differences between the groups. The difference significance levels were set at *p < 0.05, **p < 0.01, ***p < 0.001 and ****p < 0.0001. *n.s.*, no significance.

### Supplementary Information

**Additional file 1: Figure S1.** Schematic diagram of MPEG-CD synthesis. **Figure S2.** The FT-IR of MPEG-CD.** Figure S3.** ^1^H-NMR of MPEG-CD. **Figure S4.** MTX NPs in aqueous solution. **Figure S5.** ^1^H-NMR spectra of MTX, MPEG-CD and MPEG-CD@MTX (MTX NPs). **Figure S6.** 2D NOESY spectrum of MPEG-CD@MTX inclusion complex (MTX NPs). **Figure S7.** Coomassie brilliant blue bands of macrophages, MM and MM@MTX NPs. **Figure S8.** Cell viability of (A) ECs, (B) RAW 264.7 cells and (C) LPS-induced RAW 264.7 cells after incubation with free MTX, MTX NPs or MM@MTX NPs for 24 h. (***p < 0.001; *n.s.*, no significance.). **Figure S9.** Hemolysis percentage and visual images of the hemolysis test with free MTX, MTX NPs or MM@MTX NPs. **Figure S10.** Toxic effects of different concentrations of free MTX, MTX NPs and MM@MTX NPs on zebrafish embryos. **Figure S11.** Quantification of (A) IL-1β, (B) TNF-α and (C) IFN-β mRNA levels in RAW 264.7 cells by real-time PCR. (*n* = 5, *p < 0.05, **p < 0.01, ***p < 0.001; *n.s.,* no significance). **Figure S12.** The body weight change of mice during 30 days. **Figure S13.** Blood cell counts of immune-associated cells including (A) lymphocyte, (B) monocyte and (C) neutrophil. **Table S1.** Comparative ^1^H-NMR chemical shifts of (δ, ppm) studies of free MTX, β-CD, MPEG-CD@MTX inclusion complex (MTX NPs), and their complexation induced shifts (Δδ). (Δδ ppm = δ complex–δ free). (ND: no detected).

## Data Availability

All data contained in the study are in this article.
