# Supplementary material for: Biomimetic nanoparticles to enhance the reverse cholesterol transport for selectively inhibiting development into foam cell in atherosclerosis
Source: J Nanobiotechnology. 2023 Aug 29;21:307. doi: 10.1186/s12951-023-02040-9 (PMC10463892; doi:10.1186/s12951-023-02040-9)
Supplement: Supplementary file 1 — Additional file 1: Figure S1. Schematic diagram of MPEG-CD synthesis. Figure S2. The FT-IR of MPEG-CD. Figure S3. 1H-NMR of MPEG-CD. Figure S4. MTX NPs in aqueous solution. Figure S5. 1H-NMR spectra of MTX, MPEG-CD and MPEG-CD@MTX (MTX NPs). Figure S6. 2D NOESY spectrum of MPEG-CD@MTX inclusion complex (MTX NPs). Figure S7. Coomassie brilliant blue bands of macrophages, MM and MM@MTX NPs. Figure S8. Cell viability of (A) ECs, (B) RAW 264.7 cells and (C) LPS-induced RAW 264.7 cells after incubation with free MTX, MTX NPs or MM@MTX NPs for 24 h. (***p < 0.001; n.s., no significance.). Figure S9. Hemolysis percentage and visual images of the hemolysis test with free MTX, MTX NPs or MM@MTX NPs. Figure S10. Toxic effects of different concentrations of free MTX, MTX NPs and MM@MTX NPs on zebrafish embryos. Figure S11. Quantification of (A) IL-1β, (B) TNF-α and (C) IFN-β mRNA levels in RAW 264.7 cells by real-time PCR. (n = 5, *p < 0.05, **p < 0.01, ***p < 0.001; n.s., no significance). Figure S12. The body weight change of mice during 30 days. Figure S13. Blood cell counts of immune-associated cells including (A) lymphocyte, (B) monocyte and (C) neutrophil. Table S1. Comparative 1H-NMR chemical shifts of (δ, ppm) studies of free MTX, β-CD, MPEG-CD@MTX inclusion complex (MTX NPs), and their complexation induced shifts (Δδ). (Δδ ppm = δ complex–δ free). (ND: no detected). [file 12951_2023_2040_MOESM1_ESM.docx]

**Additional file for**

**Biomimetic nanoparticles to enhance the reverse cholesterol transport for selectively inhibiting development into foam cell in atherosclerosis**

Li Zhu^1^, Hongjiao Li^2^, Jiyu Li^1^, Yuan Zhong^1^, Shuai Wu^1^, Meng Yan^1^, Sheng Ni^1^, Kun Zhang^1,3^, Guixue Wang^1,4^, Kai Qu^1,3,^*, Deqin Yang^2,^*, Xian Qin^1,3,^*, Wei Wu^1,4,^*

^1.^ Key Laboratory for Biorheological Science and Technology of Ministry of Education, State and Local Joint Engineering Laboratory for Vascular Implants, Bioengineering College of Chongqing University, Chongqing, 400044, China

^2.^ School and Hospital of Stomatology, Chongqing Medical University, Chongqing, 404100, China

^3.^ Chongqing University Three Gorges Hospital, Chongqing, 404000, China.

^4.^ Jin Feng Laboratory, Chongqing, 401329, China.

***Corresponding authors:**

david2015@cqu.edu.cn (Wei Wu);[qinxian224@cqu.edu.cn(Xian](mailto:qinxian224@cqu.edu.cn(Xian) Qin)

yangdeqin@hospital.cqmu.edu.cn (Deqin Yang); [qukaigood@qq.com](mailto:qukaigood@qq.com) (Kai Qu);

**This file includes:**

**Figures S1~S12**

**Tables S1**

**Supporting Figures**

**
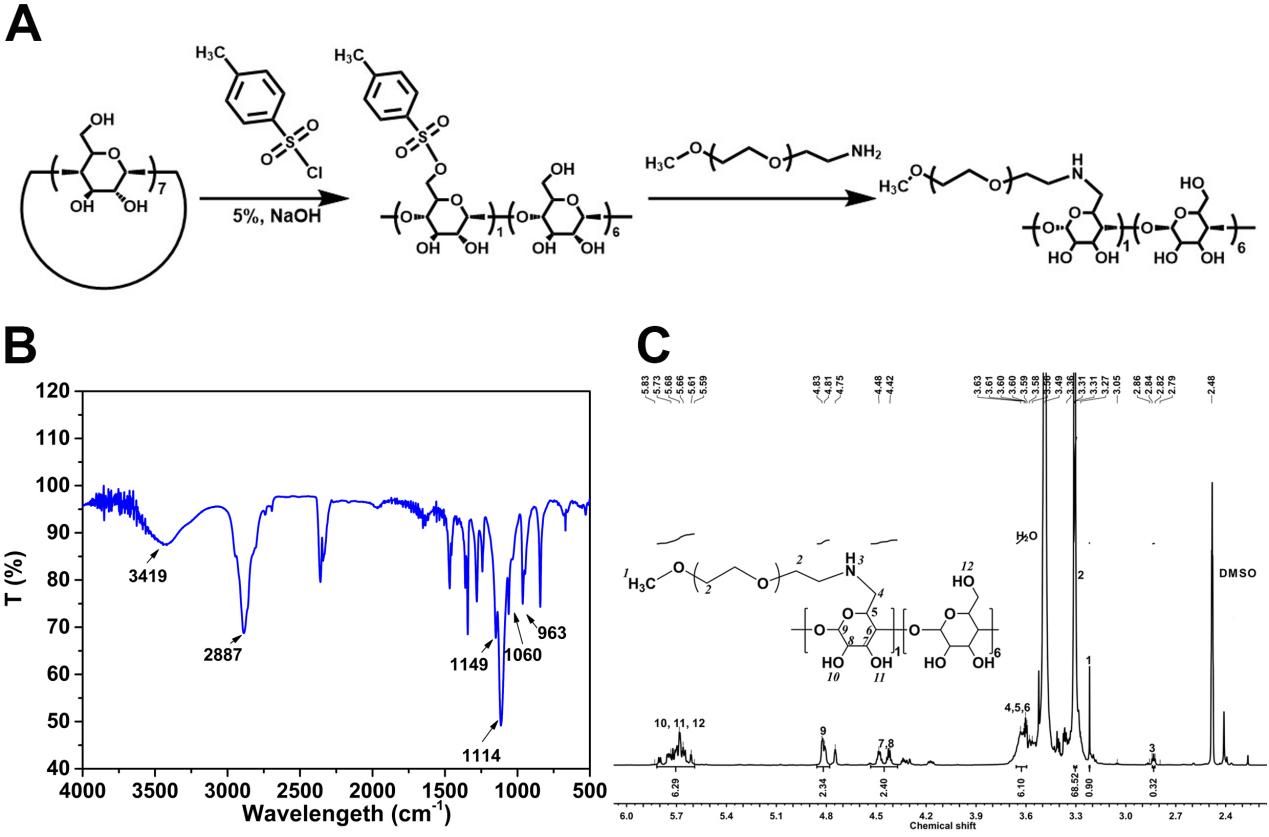
**

**Figure. S1.** Schematic diagram of MPEG-CD synthesis.





**Figure. S2.** The FT-IR of MPEG-CD.


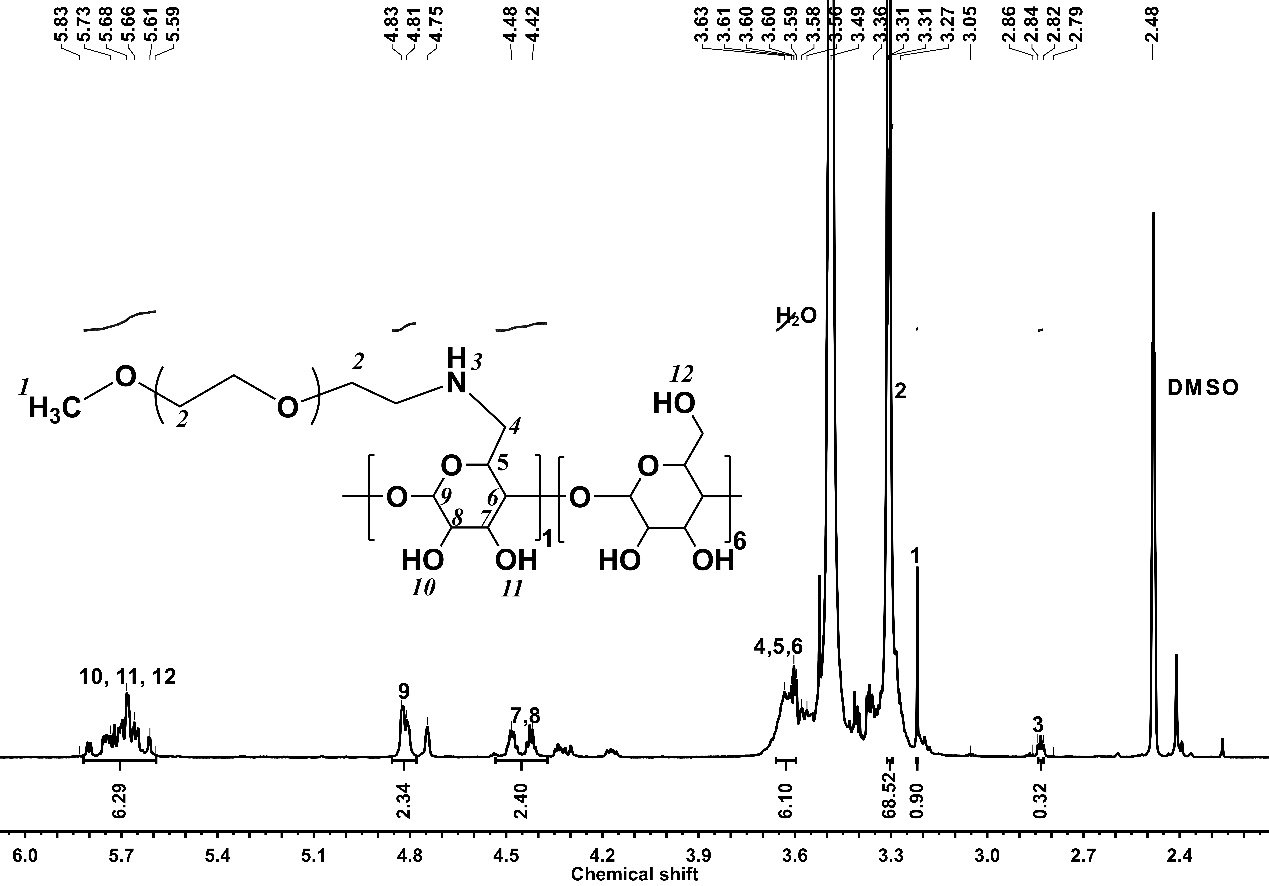


**Figure. S3.** ^1^H-NMR of MPEG-CD.


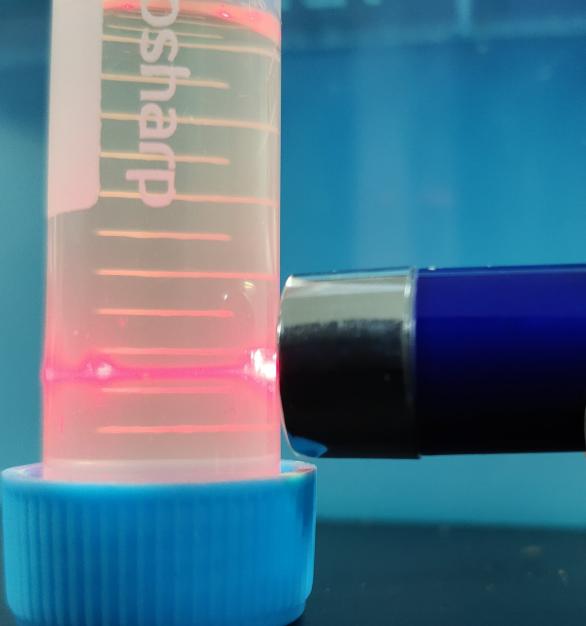


**Figure. S4.** MTX NPs in aqueous solution.


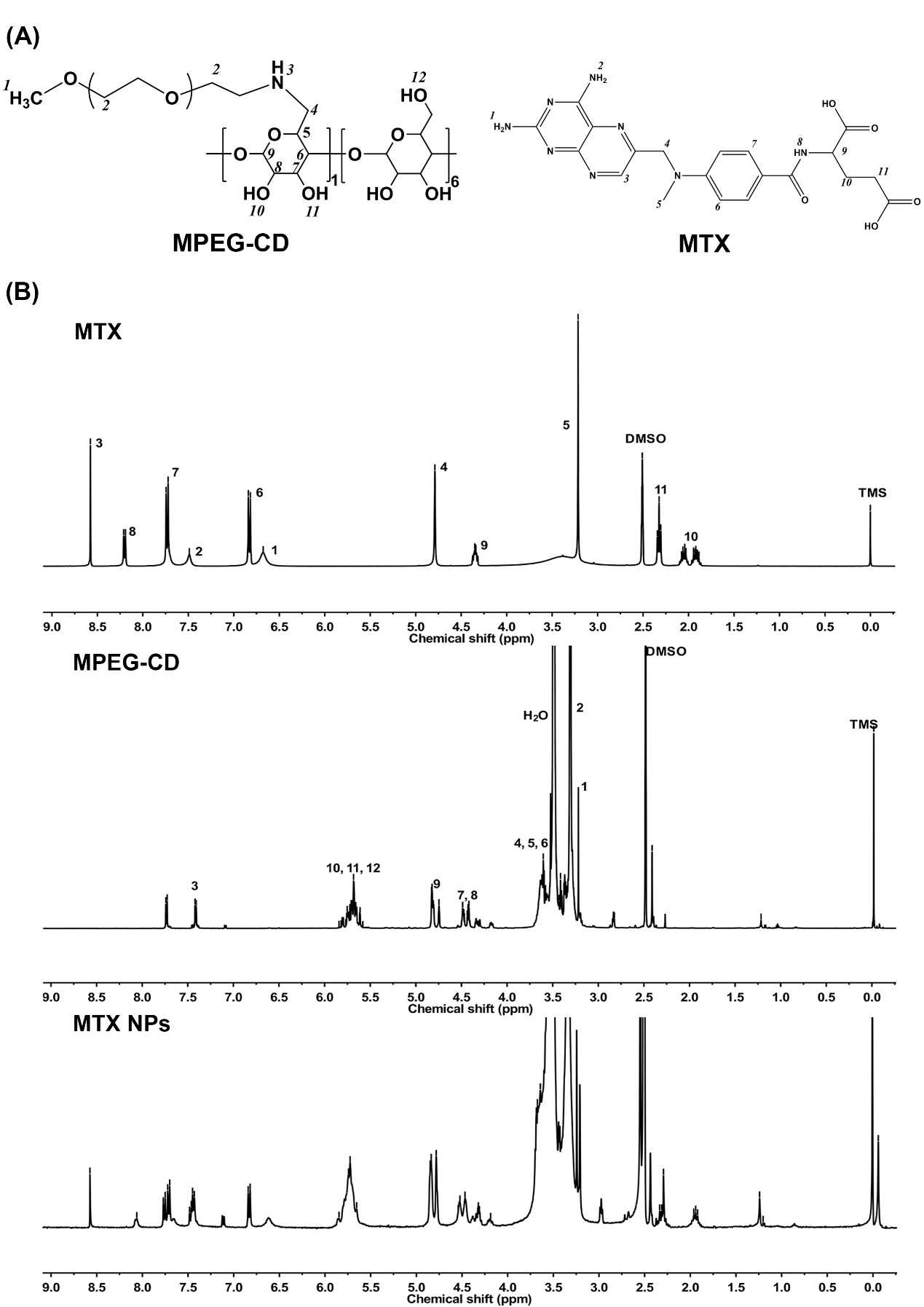


**Figure. S5.** ^1^H-NMR spectra of MTX, MPEG-CD and MPEG-CD@MTX (MTX NPs).

**Table. S1.** Comparative ^1^H-NMR chemical shifts of (δ, ppm) studies of free MTX, β-CD, MPEG-CD@MTX inclusion complex (MTX NPs), and their complexation induced shifts (Δδ). (Δδ ppm = δ complex–δ free). (ND: no detected)

| H Protons | δ(β-CD) | δ(MTX NPs) | Δδ (ppm) | H Protons | δ(MTX) | δ(MTX NPs) | Δδ (ppm) |
| --- | --- | --- | --- | --- | --- | --- | --- |
| H-4 | 3.60 | 3.63 | 0.03 | H-1 | 6.68 | 6.63 | -0.05 |
| H-5 | 3.35 | 3.42 | 0.07 | H-2 | 7.49 | 7.45 | -0.04 |
| H-6 | 3.52 | ND | ND | H-3 | 8.58 | 8.58 | 0 |
| H-7 | 4.48 | 4.49 | 0.01 | H-4 | 4.79 | 4.78 | -0.01 |
| H-8 | 4.41 | 4.42 | 0.01 | H-5 | 3.21 | 3.21 | 0 |
| H-9 | 4.83 | 4.83 | 0 | H-6 | 6.83 | 6.82 | -0.01 |
|  |  |  |  | H-7 | 7.73 | 7.72 | -0.01 |
|  |  |  |  | H-8 | 8.21 | 8.06 | -0.15 |
|  |  |  |  | H-9 | 4.34 | 3.34 | 0 |
|  |  |  |  | H-10 | 2.04 | 1.94 | -0.1 |
|  |  |  |  | H-11 | 2.32 | 2.31 | -0.01 |


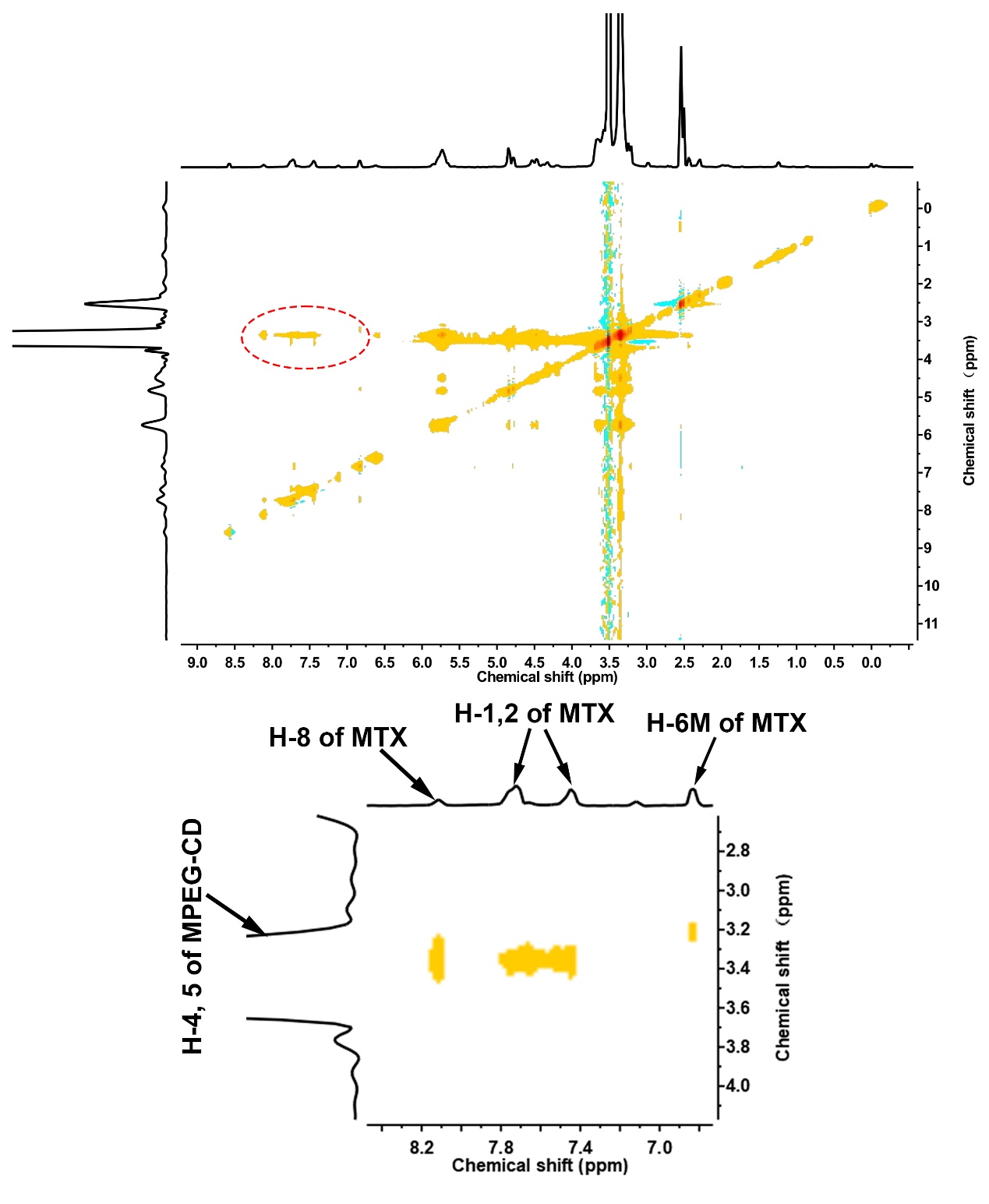


**Figure. S6.** 2D NOESY spectrum of MPEG-CD@MTX inclusion complex (MTX NPs).


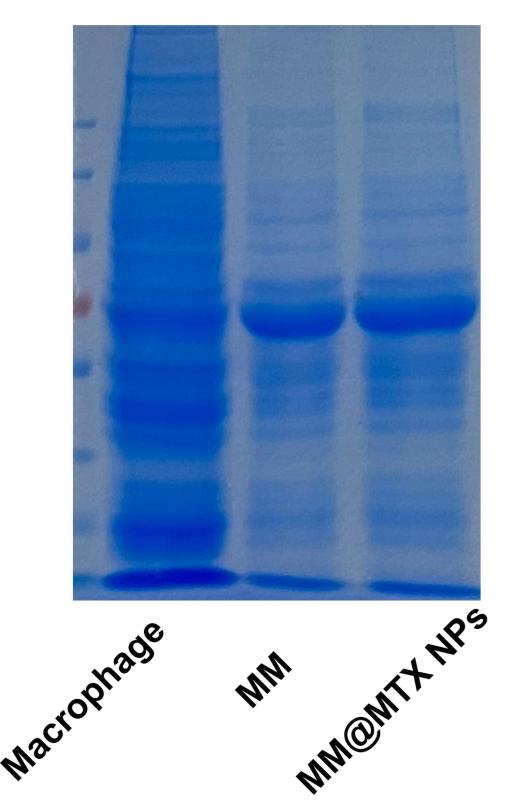


**Figure. S7.** Coomassie brilliant blue bands of macrophages, MM and MM@MTX NPs.

**
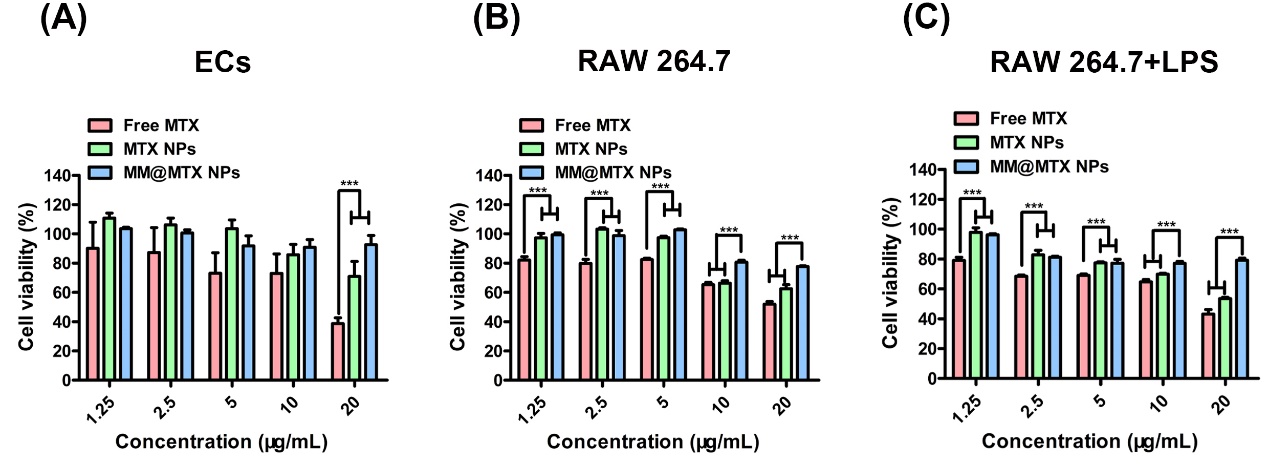
**

**Figure. S8.** Cell viability of (A) ECs, (B) RAW 264.7 cells and (C) LPS-induced RAW 264.7 cells after incubation with free MTX, MTX NPs or MM@MTX NPs for 24 h. (****p* < 0.001; *n.s.*, no significance.)

**
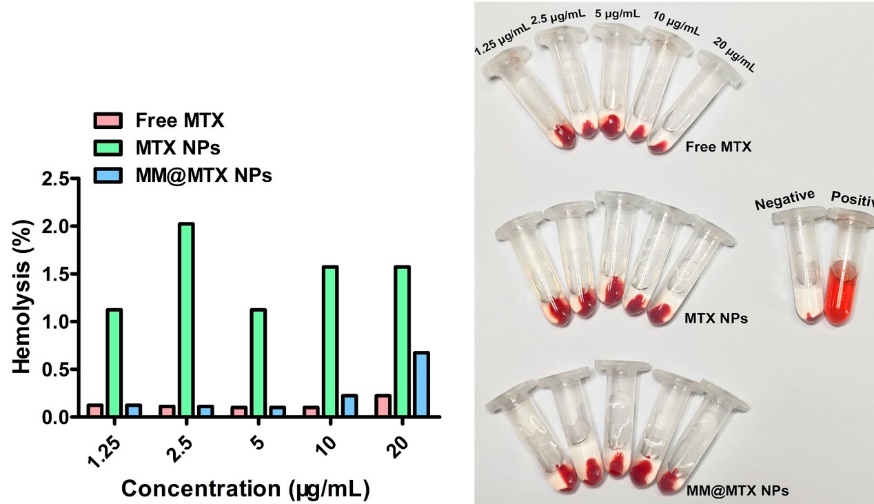
**

**Figure. S9.** Hemolysis percentage and visual images of the hemolysis test with free MTX, MTX NPs or MM@MTX NPs.


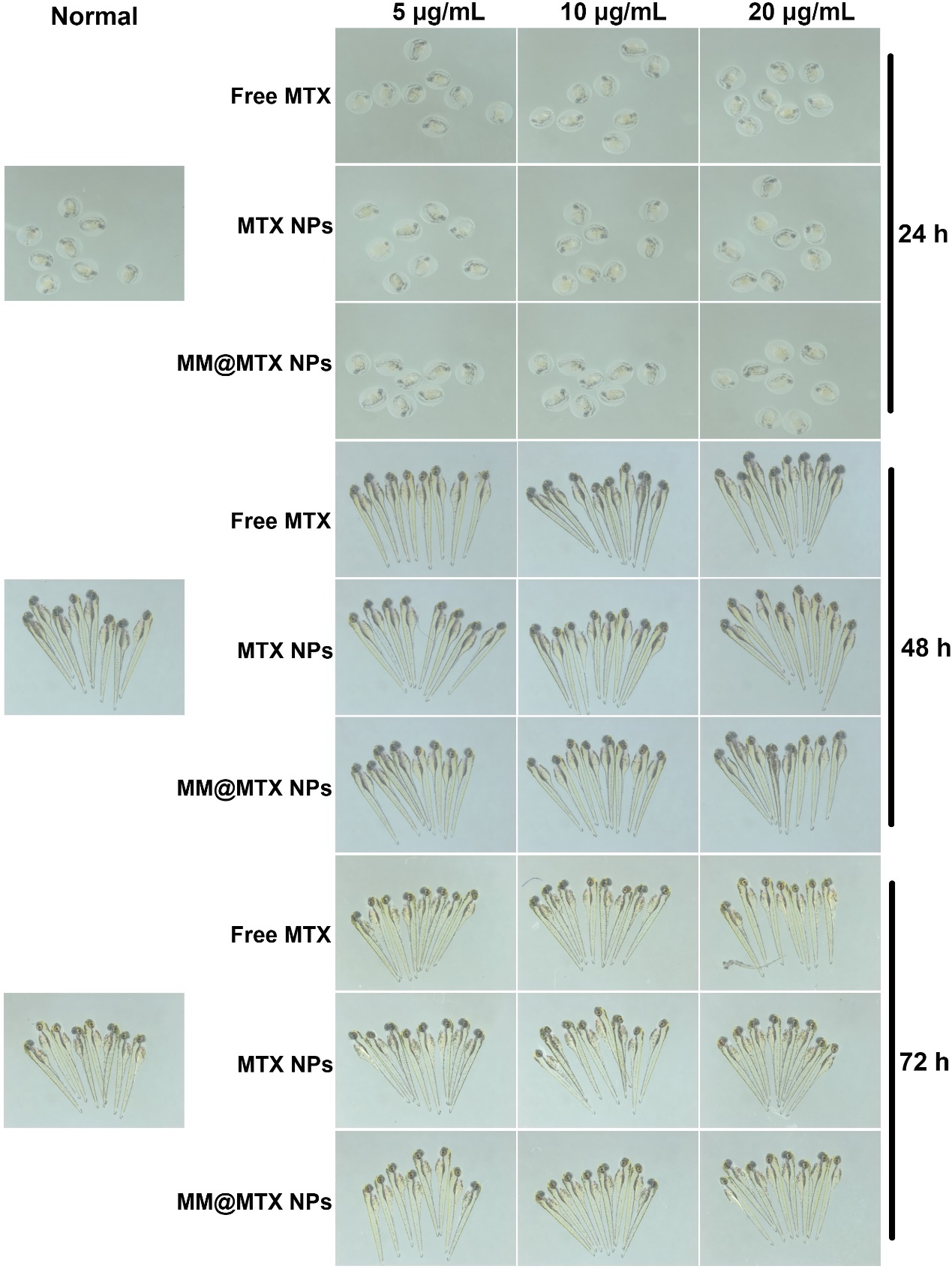


**Figure. S10.** Toxic effects of different concentrations of free MTX, MTX NPs and MM@MTX NPs on zebrafish embryos.


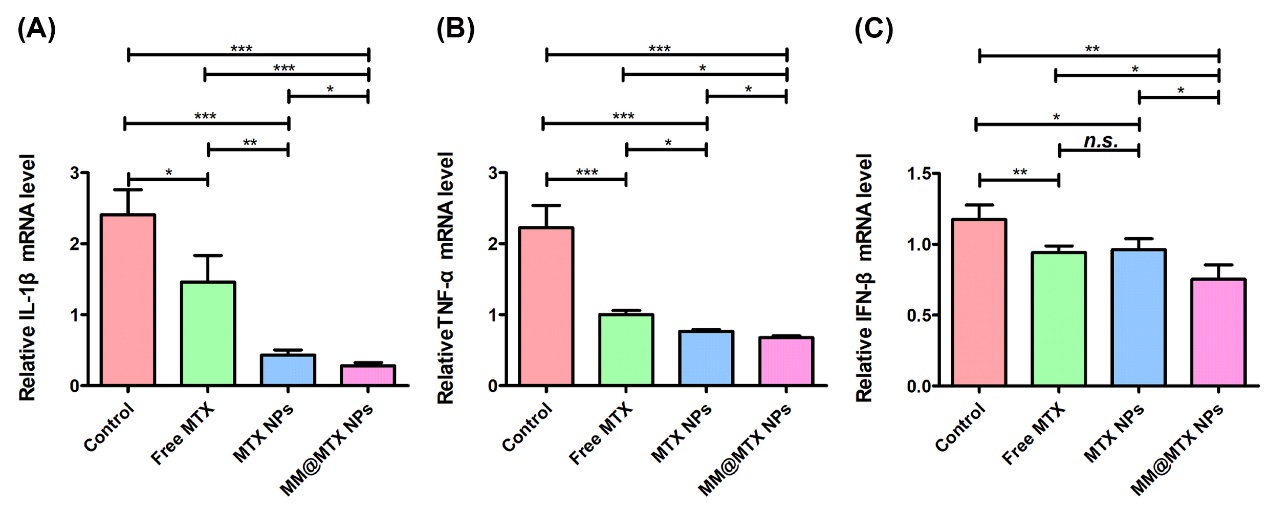


**Figure. S11.** Quantification of (A) IL-1β, (B) TNF-α and (C) IFN-β mRNA levels in RAW 264.7 cells by real-time PCR. (*n* = 5, **p* < 0.05, ***p* < 0.01, ****p* < 0.001; *n.s.,* no significance)





**Figure. S12.** The body weight change of mice during 30 days.


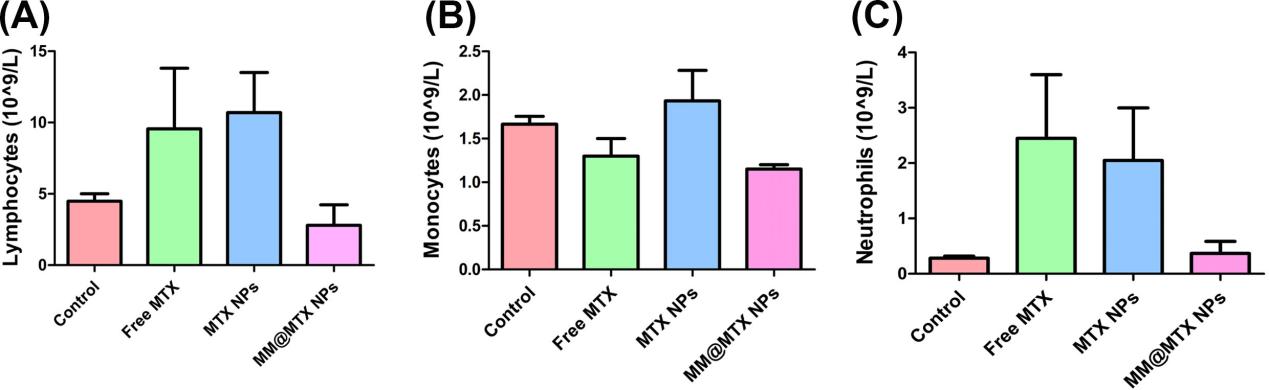


**Figure. S13.** Blood cell counts of immune-associated cells including (A) lymphocyte, (B) monocyte and (C) neutrophil.
